# Mapping the Human miRNA Interactome by CLASH Reveals Frequent Noncanonical Binding

**DOI:** 10.1016/j.cell.2013.03.043

**Published:** 2013-04-25

**Authors:** Aleksandra Helwak, Grzegorz Kudla, Tatiana Dudnakova, David Tollervey

**Affiliations:** 1 Wellcome Trust Centre for Cell Biology, The University of Edinburgh, Edinburgh EH9 3JR, UK

## Abstract

MicroRNAs (miRNAs) play key roles in gene regulation, but reliable bioinformatic or experimental identification of their targets remains difficult. To provide an unbiased view of human miRNA targets, we developed a technique for ligation and sequencing of miRNA-target RNA duplexes associated with human AGO1. Here, we report data sets of more than 18,000 high-confidence miRNA-mRNA interactions. The binding of most miRNAs includes the 5′ seed region, but around 60% of seed interactions are noncanonical, containing bulged or mismatched nucleotides. Moreover, seed interactions are generally accompanied by specific, nonseed base pairing. 18% of miRNA-mRNA interactions involve the miRNA 3′ end, with little evidence for 5′ contacts, and some of these were functionally validated. Analyses of miRNA:mRNA base pairing showed that miRNA species systematically differ in their target RNA interactions, and strongly overrepresented motifs were found in the interaction sites of several miRNAs. We speculate that these affect the response of RISC to miRNA-target binding.

## Highlights

• A experimental approach identifies in vivo targets for miRNAs
• miRNAs show frequent noncanonical targeting of mRNAs
• Nonseed interactions are common and functional
• miRNA base pairing shows distinct patterns and overrepresented motifs

## Graphical Abstract

**Figure:**
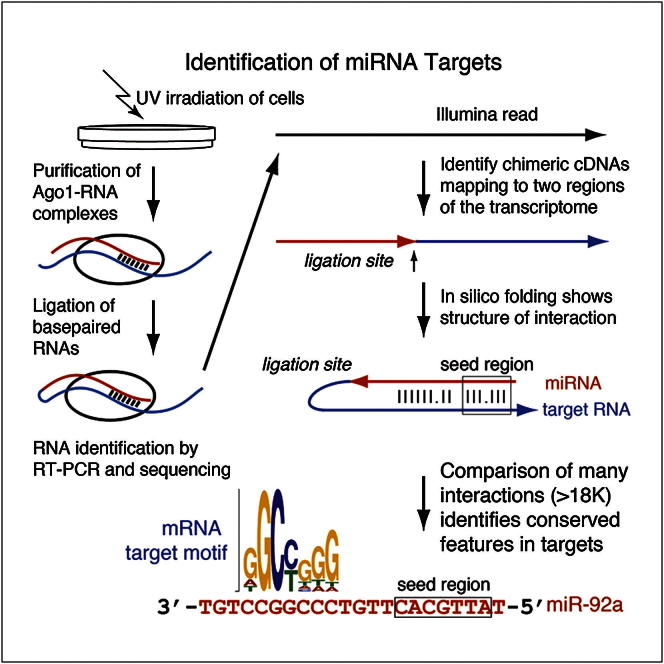

## Teaser

The CLASH approach identifies more than 18,000 interactions between miRNA and mRNA molecules in human cells, revealing many noncanonical interactions involving bulged or mismatched nucleotides, as well as interactions outside the proposed 5′ “seed” region of miRNAs.

## Introduction

MicroRNAs (miRNAs) play a key role in the posttranscriptional regulation of gene expression by guiding the association between the RNA-induced silencing complex (RISC) and target RNAs (reviewed in Fabian et al., 2010). Human cells express more than 1,000 miRNAs, each potentially binding to hundreds of messenger RNAs (mRNAs) (Lewis et al., 2005), but only a small fraction of these interactions has been validated experimentally. Experiments conducted throughout the last decade have established a set of canonical rules of miRNA-target interactions (reviewed in Bartel, 2009): (1) interactions are mediated by the “seed” region, a 6- to 8-nt-long fragment at the 5′ end of the miRNA that forms Watson-Crick pairs with the target; (2) nucleotides paired outside the seed region stabilize interactions but are reported not to influence miRNA efficacy (Garcia et al., 2011; Grimson et al., 2007); and (3) functional miRNA targets are localized close to the extremes of the 3′ UTRs of protein-coding genes in relatively unstructured regions (Grimson et al., 2007). Recently, RISC-binding sites on mRNAs have been mapped transcriptome wide by crosslinking, immunoprecipitation, and high-throughput sequencing (CLIP-seq), allowing prediction of many miRNA-mRNA interactions (Chi et al., 2009; Hafner et al., 2010a; Zhang and Darnell, 2011) and yielding data consistent with the canonical rules.

However, there is substantial evidence for exceptions to these rules. As examples, in *C. elegans*, the well-studied *lin-4::lin-14* interaction involves bulged nucleotides (Ha et al., 1996), whereas the *let-7::lin-41* interaction involves wobble G·U pairing (Vella et al., 2004). Human miR-24 targets important cell-cycle genes using interaction sites that are spread over almost the whole miRNA. These interactions lack obvious seed pairing and contain multiple mismatches, bulges, and wobbles (Lal et al., 2009). Analysis of the miR-124 targets recovered by HITS-CLIP revealed a mode of miRNA-mRNA binding that involves a G bulge in the target, opposite miRNA nucleotides 5 and 6. It has been estimated that about 15% of miR-124 targets in mice brain are recognized by this mode of binding (Chi et al., 2012). Another, apparently rare, base-pairing pattern called “centered site” (Shin et al., 2010) involves 11 consecutive Watson-Crick base pairs between the target and positions 4–14 or 5–15 of miRNA. There are also multiple exceptions regarding the requirement for miRNA-binding sites to be located in the 3′ UTR. Functional miRNA-binding sites have occasionally been reported in 5′ UTRs (Grey et al., 2010) and, more frequently, within mRNA coding sequences (Hafner et al., 2010a; Reczko et al., 2012). Moreover, recent reports show that miRNA targets are not limited to protein-coding transcripts and can be found in noncoding RNAs (ncRNAs) that arise from pseudogenes (Poliseno et al., 2010). Together, these data indicate that miRNAs can bind to a wide variety of targets, with both canonical and noncanonical base pairing, and indicate that miRNA targeting rules may be complex and flexible.

To allow direct, high-throughput mapping of RNA-RNA interactions, we previously developed crosslinking, ligation, and sequencing of hybrids (CLASH) (Kudla et al., 2011). High-throughput methods have been developed to map protein-DNA interactions, protein-RNA interactions, and DNA-DNA interactions, so CLASH completes the toolkit necessary to study nucleic acid interactomes. Here, we adapted CLASH to allow direct observation of miRNA-target pairs as chimeric reads in deep-sequencing data. Our transcriptome-wide data set reveals the prevalence of seed and nonseed interactions and the diversity of in vivo targets for miRNAs.

## Results

### CLASH Directly Maps miRNA-Binding Sites

**Figure 1:**
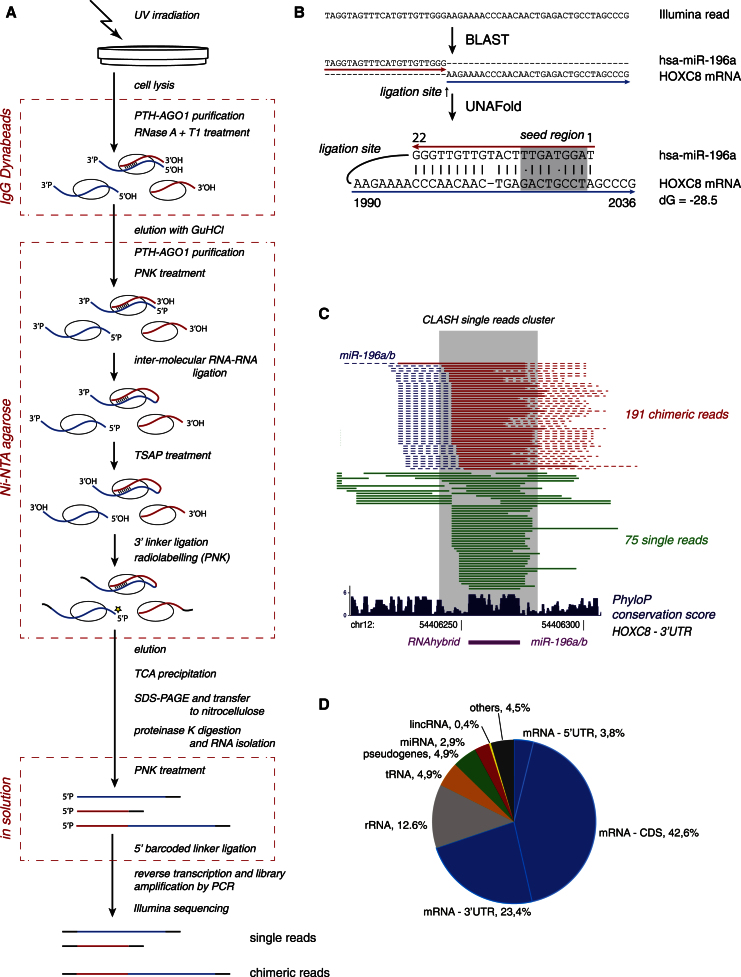
Overview of Experimental and Bioinformatic Procedures (A) Growing cells were UV irradiated, and PTH-AGO1 was purified. RNA fragmentation, ligation, cDNA synthesis, and sequencing of AGO1-associated RNAs allowed the identification of sites of AGO1 binding (as single reads) and RNA-RNA interactions at AGO1-binding sites (as chimeric reads). (B) Sequencing reads were mapped to a database of human transcripts using BLAST (Altschul et al., 1990). Sequences reliably mapped to two different sites were folded in silico using UNAFold (Markham and Zuker, 2008) to identify the interaction site of the RNA molecules that gave rise to the chimeric cDNA. (C) Example interaction between miR-196a/b and *HOXC8* that was supported by chimeric reads (red), and a cluster of nonchimeric reads (green). The blue dashed line represents the location of the miRNA bit of chimera, and the red dashed line shows the 25 nt mRNA extension added during the analysis. The interaction was previously shown experimentally (Li et al., 2010) and can be predicted by RNAhybrid (Rehmsmeier et al., 2004). (D) Distribution of all miRNA interactions among various classes of RNAs. The main miRNA targets are mRNAs and are represented by 18,514 interactions. See also Figure S1 and Tables S1 and S2A–S2C.

To recover RNA species bound to the human RISC complex, we created an N-terminal fusion of hAGO1 with a protein A-TEV cleavage site-His_6_ tripartite tag (PTH-AGO1). N-terminally tagged AGO proteins were used previously in many studies and were shown to be functional (Chatterjee and Grosshans, 2009; Lian et al., 2009). Actively growing Flp-In T-REx 293 cells stably expressing PTH-AGO1 were UV irradiated (254 nm) to crosslink proteins to interacting RNAs. PTH-AGO1 was purified, and interacting RNA molecules were partially hydrolyzed, ligated, reverse transcribed, and subjected to Illumina sequencing. At the ligation step, RNA molecules present in AGO-associated miRNA-target duplexes can be joined together (Figure 1A). Following RT-PCR amplification, these generate “chimeric” complementary DNAs (cDNAs), which can be identified because they contain two regions that map to sites that are noncontiguous in the transcriptome sequence (Figure 1B).

When AGO1-associated RNAs were analyzed, around 98% were “single reads” representing AGO1-binding sites on RNAs, similar to those obtained with HITS-CLIP and PAR-CLIP (Chi et al., 2009; Hafner et al., 2010a). However, ∼2% were chimeric reads reflecting intermolecular stem structures present in the AGO1-associated RNAs (Figures 1A and 1B). Supporting the significance of the chimeras, 94% of the sequences involved were also recovered as single reads in at least one experiment (Figure 1C). As a control experiment, the lysate obtained from UV-irradiated human cells was mixed with an equal quantity of yeast lysates prior to CLASH analysis (details in Extended Experimental Procedures and Table S2C). This revealed that the background arising from RNA-RNA interactions formed in vitro represents <2% of single and chimeric reads, confirming that interactions recovered by CLASH were predominately formed in vivo.

**Figure S1:**
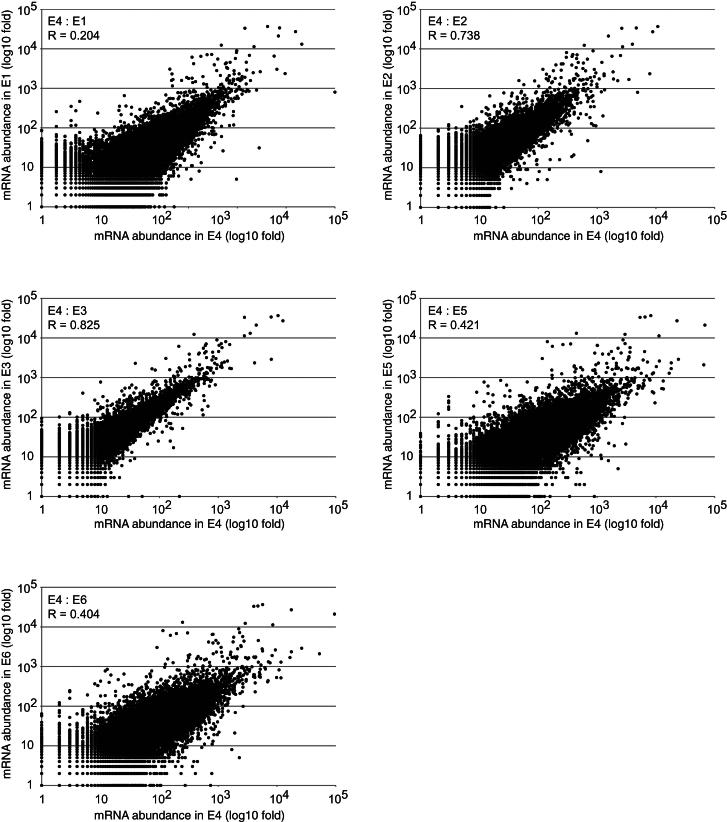
Comparison of CLASH Data Sets E1–E6, Related to Figure 1 The graphs present the number of reads mapped to each mRNA in experiment E4 relative to the other experiments. R – Pearson’s correlation coefficient.

Six independent experiments (E1–E6) were performed with slightly differing protocols, yielding broadly comparable data that were analyzed together (Figure S1 and Tables S1 and S2A available online). mRNAs form the principal class of miRNA binding partners identified in chimeric reads and constitute nearly 70% of interactions (Figure 1D). Other known target classes were recovered, including pseudogenes and long-intergenic ncRNAs (lincRNAs), as were substantial numbers of chimeras with ribosomal RNAs (rRNAs), transfer RNAs (tRNAs), small nuclear RNAs (snRNAs), and miRNAs. The 18,514 miRNA-mRNA interactions identified from chimeric reads were analyzed in detail. These represent 399 different miRNAs and 6,959 different protein-coding genes (Table S2B). The full set of miRNA-mRNA chimeras identified is included in Data S1.

### Validation of Interactions Identified by CLASH Supports Their Reliability

To assess whether the interactions identified are functionally important, we determined whether they (1) resemble known and predicted miRNA-mRNA interactions, (2) show evolutionary conservation, and (3) are associated with downregulation of target genes.

The CLASH data set included a number of previously known interactions (Table S3). For example, the association between miR-196a/b and transcripts from the *HOX* gene family (*HOXB8* and *HOXC8*) (Yekta et al., 2004) (Figure 1C) was found in five of six CLASH experiments and was supported by 275 chimeric reads. In addition to the known interaction in the 3′ UTR of *HOXC8*, we identified a miR-196 interaction in the 5′ UTR. In contrast, interactions involving liver-specific miR-122 or brain-specific miR-124 were strongly depleted, highlighting the tissue specificity of the miRNA interactome recovered by CLASH from HEK cells.

To estimate the overlap between the CLASH targets (i.e., interactions identified in miRNA-mRNA chimeras) and experimentally determined AGO-binding sites in mRNAs, we compared chimeras and single reads from the present study. 94% of CLASH targets were identified as AGO1-binding sites in the nonchimeric reads; 3,066 of these (16.6% of all) were high-confidence clusters of 20 or more distinct nonchimeric reads. In addition, 1,596 CLASH targets coincided with high-confidence AGO1-4-binding sites previously mapped by PAR-CLIP in HEK293 cells (Hafner et al., 2010a), a 3-fold enrichment over expected chance levels for expression-matched transcripts (Table S4A). CLASH targets were also compared to sets of miRNA targets bioinformatically predicted by the programs miRanda (John et al., 2004), PicTar (Krek et al., 2005), PITA (Kertesz et al., 2007), RNAhybrid (Rehmsmeier et al., 2004), and TargetScan (Lewis et al., 2005) (Table S4B). This analysis was limited to CLASH targets located in 3′ UTRs of human RefSeq transcripts because published predictions are generally restricted to these regions. CLASH targets were highly enriched (average 14-fold) in the predicted data compared to controls. These findings strongly indicate that chimeras faithfully reflect in vivo miRNA-mRNA interactions.

**Figure 2:**
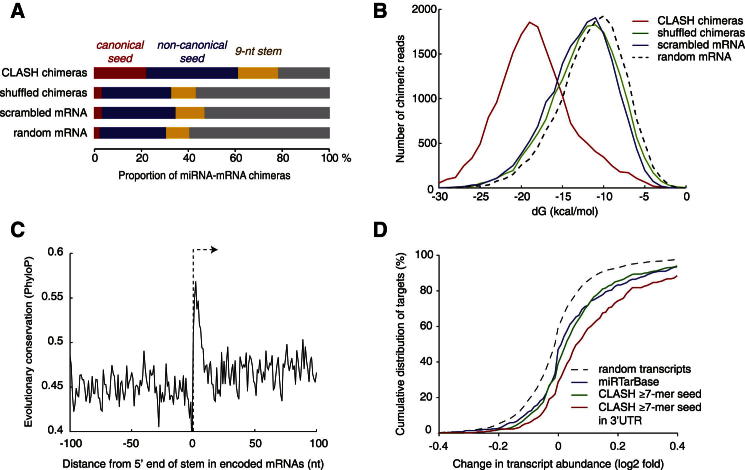
Bioinformatic and Experimental Validation of miRNA-mRNA Interactions (A) Proportion of canonical seed interactions (exact Watson-Crick pairing of nts 2–7 or 3–8 of the miRNA), noncanonical seed interactions (pairing in positions 2–7 or 3–8, allowing G-U pairs and up to one bulged or mismatched nucleotide), or 9 nt stems (allowing bulged nucleotides in the target) among CLASH chimeras and several randomized data sets; the differences between CLASH and randomized data sets were highly significant (chi-square tests, p < 10^−300^, p < 10^−100^, and p < 10^−80^ for canonical seeds, noncanonical seeds, and stems, respectively). (B) The mean predicted binding energy between miRNA and matching target mRNA found in chimeras was stronger by over 5 kcal mol^−1^ than in randomly matched pairs (t test, p < 10^−300^). (C) Average conservation score along mRNA 3′ UTRs, centered at the 5′ end of the longest stem predicted within each CLASH target. The mean conservation score within predicted stems was significantly higher than in flanking regions of the 3′ UTR (0.54 versus 0.46, t test, p < 10^−26^, n = 4634). (D) Changes in mRNA abundance following the depletion of 25 miRNAs (Hafner et al., 2010a). The graph shows a cumulative distribution of the log2 fold change (LFC) of mRNA abundance upon miRNA depletion for different sets of mRNAs: targets of the 25 miRNAs identified by CLASH with a 7-mer seed match (green line), CLASH targets in the 3′ UTR with 7-mer seed match (red line), targets extracted from the miRTarBase (blue line), and random transcripts with expression levels matching the CLASH targets (dashed line). Displacement of the curve to the right reveals increased abundance following miRNA depletion, which is indicative of mRNA repression in the presence of the tested miRNAs. See also Figure S2; Tables S3, S4A, and S4B; and Data S1.

Many characterized miRNA interactions involve perfect complementarity between the miRNA 5′ region, particularly nucleotides 2–8 (known as the seed sequence) and the target RNA. Comparison to randomized sequences showed strong enrichment for exact (Watson-Crick, “canonical seed”) and near-exact (G·U pairs, up to one nt mismatch or bulge; “noncanonical seed”) 6-mer seed matches among chimeras (Figure 2A). Notably, noncanonical seed interactions were ∼1.7-fold more common than perfect base pairing.

**Figure S2:**
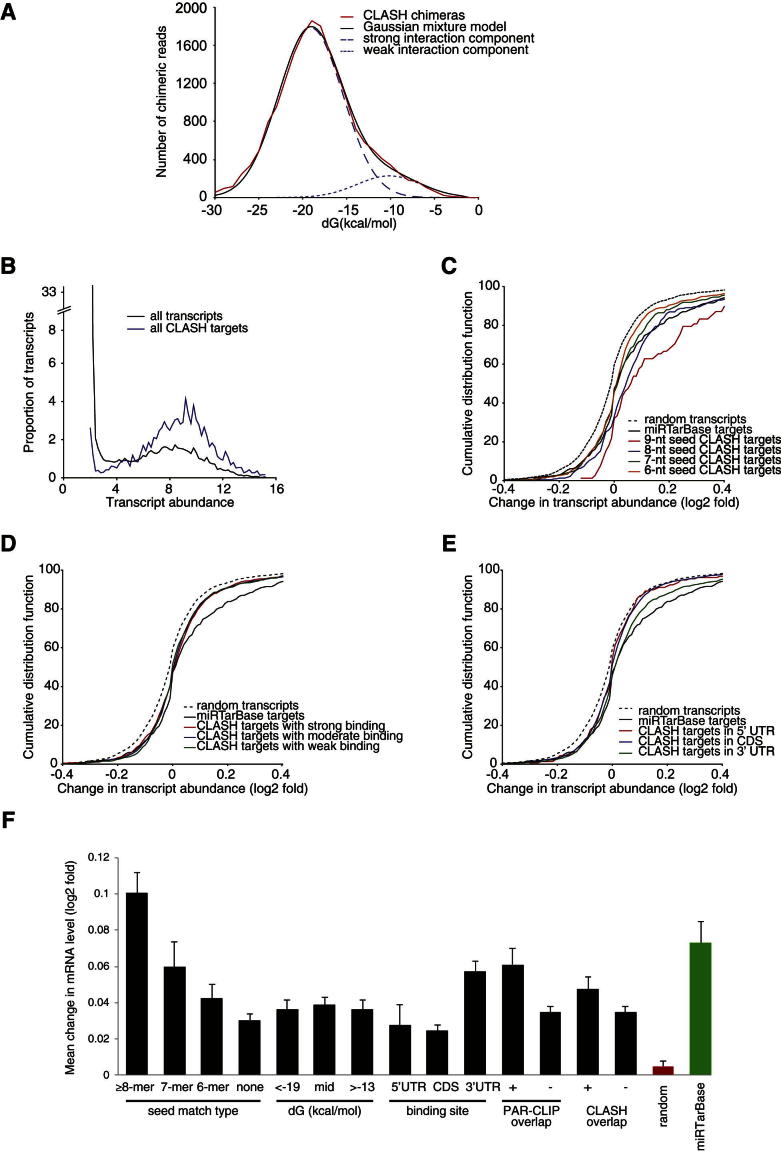
Validation of miRNA Targets, Related to Figure 2 (A) Distribution of predicted miRNA-mRNA interaction stability. A Gaussian mixture model (black line) fitted to the observed distribution of folding energies in the chimeric sequences recovered (red line) using the least-squares method. The weak-interaction component (11% of interactions, dotted blue line) is similar to random interactions (see Figure 2B). Comparison to the distribution of different classes of miRNA-mRNA interactions recovered in individual CLASH experiments (see Figure S3, below) indicates that the weak-interaction component is the least reproducible and is therefore likely to have a higher contribution of noise (for example, random ligation between RNA fragments in solution, or reverse transcriptase template switching events). The remaining 89% of the observed chimeras show stronger folding (dashed blue line), most probably because they predominately originate from genuine miRNA-mRNA hybrids. (B–F) Transcriptome-wide changes in the levels of mRNAs identified as miRNA targets by CLASH were assessed by retrospective analysis of the reported effects of simultaneous depletion of 25 different miRNAs (Hafner et al., 2010a). The mRNAs were filtered by the location and nature of the miRNA-mRNA interactions identified by CLASH. (B) CLASH identifies miRNA targets among transcripts of various abundance levels. (C) Changes in mRNA abundance following the depletion of 25 miRNAs are shown for: Known targets of these miRNAs downloaded from miRTarBase, for CLASH targets with different lengths of k-mer seed matches with any of the 25 miRNAs and for a set of random transcripts with expression levels similar to the CLASH targets. (D) CLASH targets were categorized according to their predicted binding energy with miRNA; strong binding, dG < −19.4 kcal/mol; weak binding, dG > −13.4 kcal/mol. (E) CLASH targets were categorized according to the position of their binding site in the transcript. (F) Mean changes in transcript abundance for CLASH targets categorized by seed match length, location, predicted binding energy, and presence or absence of overlap with AGO binding clusters from PAR-CLIP (Hafner et al., 2010a) or CLASH (present study). Error bars represent standard error. All classes of targets, except the 5′ UTR class, are significantly different from random (p < 0.05, Wilcoxon rank sum test with Bonferroni correction).

Binding energies for the miRNA-mRNA interactions were predicted from in silico folding of sequences recovered in chimeras and compared to predicted binding energies in several control data sets (Figures 1B and 2B). This showed that miRNA-mRNAs chimeras recovered are strongly enriched for stably base-paired interactions. The strong binding energies of chimeric reads indicate that these result from genuine RNA-RNA interactions rather than from proximity-induced ligation of noninteracting RNAs in solution. Fitting a Gaussian mixture model to the observed distribution of binding energies (Figure S2A) suggested the existence of two populations; 89% of miRNA-mRNAs duplexes recovered having a lower energy distribution than the remaining 11%. Weak interactions may be disfavored in the recovered chimeras due to loss during sample preparation. However, exact interactions are typically slightly stronger than near-exact interactions (−19.4 kcal mol^−1^ versus −18.6 kcal mol^−1^), so any bias in the CLASH method will favor exact interactions. Thus, the expected direction of bias does not explain the high numbers of near-exact interactions identified.

Evolutionary conservation has been widely used to identify miRNA-binding sites. To quantify the conservation of putative miRNA-target interactions identified by CLASH, we analyzed PhyloP conservation scores (Pollard et al., 2010) within targets mapped to 3′ UTRs of annotated mRNAs from 46 vertebrate genomes. The identified miRNA target sites showed marked conservation relative to flanking regions, supporting their biological importance (Figure 2C). Because the CLASH technique depends on the recovery and sequencing of crosslinked RNA, the results will be biased by transcript abundance. Comparison of the distribution of CLASH targets to mRNA abundance (Figure S2B) revealed enrichment for more abundant targets, as expected. However, even relatively low abundance targets are well represented in the data set, showing that the CLASH approach is not limited to abundant mRNAs.

Interactions with miRNAs frequently result in downregulation of target mRNAs. To functionally validate CLASH targets, we reanalyzed published data reporting the effects of simultaneous depletion of 25 different miRNAs on mRNA levels (Hafner et al., 2010a). The expectation is that miRNA depletion will increase the abundance of target RNAs due to loss of repression. Cognate miRNA-mRNA pairs identified by CLASH and represented in the miRNA depletion data set were retrospectively analyzed and compared to confirmed miRNA-mRNA pairs from miRTarBase (Hsu et al., 2011). Similar upregulation was observed among the CLASH targets with a canonical 7-mer seed and validated miRTarBase targets (Figure 2D). In agreement with previous findings, upregulation was highest among those targets that contained a seed match and were located in the 3′ UTR (Figures 2D and S2C–S2F). Targets lacking a canonical seed match were also upregulated, on average half as efficiently as the seed-containing targets (Figure S2F). Such interactions would not generally be identified by target prediction programs, which are biased toward canonical seed interactions, whereas our findings support their reliability. Targets in CDSs were significantly upregulated upon miRNA depletion (p = 3.4 × 10^−10^), and upregulation of sites in the CDS is about half of that in 3′ UTRs (Figure S2F). Comparisons across all predicted interactions did not reveal a clear correlation between predicted binding energy and target regulation (Figure S2D). Notably, cohorts of predicted miRNA-mRNA interactions outperform the experimentally confirmed interactions taken from miRTarBase when compared transcriptome wide for their effects on mRNA stability (Figures 2D and S2C).

### Analysis of miRNA-mRNA Base-Pairing Patterns Reveals the Prevalence of Nonseed Interactions

**Figure 3:**
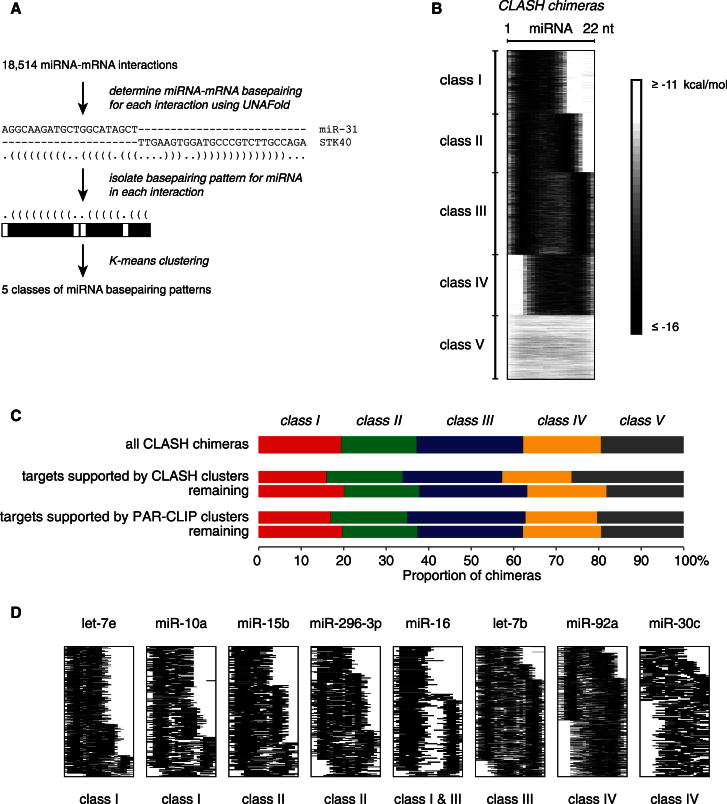
Base-Pairing Patterns in miRNA-mRNA Interactions (A) Outline of the analysis of miRNA-mRNA base-pairing patterns. Unpaired nucleotides are in white, and paired nucleotides are in shades of gray depending on the overall interaction strength. (B) Positions of base-paired nucleotides in miRNAs among the 18,514 miRNA-mRNA interactions. The names of interaction classes (I–V) are indicated. (C) Distribution of CLASH targets among the five base-pairing classes. A similar proportion of CLASH targets from each class are supported by experimentally determined AGO-binding sites, as identified by CLASH single read clusters and PAR-CLIP clusters. (D) Examples of miRNAs with nonrandom distribution across interaction classes. Of the 68 miRNAs tested, 31 were nonrandomly distributed across four classes of interaction (p < 0.05, chi-square test with Bonferroni correction; class V interactions were excluded from this analysis). See also Figure S3 and Data S2.

**Figure S3:**
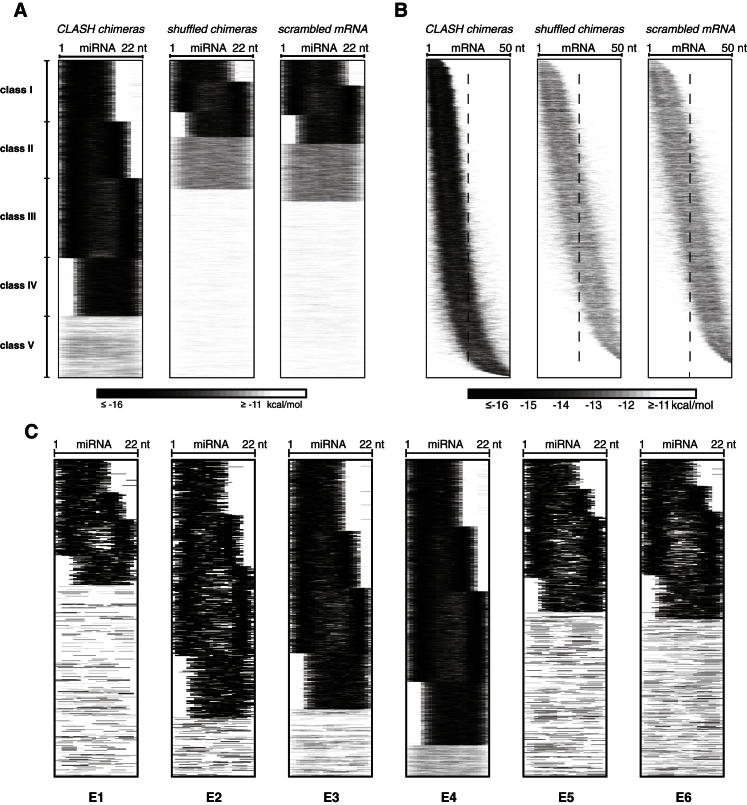
Patterns of miRNA Interactions with mRNAs, Related to Figure 3 (A) Location of base-paired nucleotides in miRNAs. For each miRNA-mRNA interaction, the minimum energy structure was determined using the hybrid-min program (Markham and Zuker, 2008). Unpaired nucleotides are shown in white and paired nucleotides are in shades of gray depending on the overall interaction strength. Similar interactions were grouped together by K-means clustering (K = 5). Left, interactions identified by CLASH; center, interactions between randomly reassigned (shuffled) miRNA-mRNA pairs; right, interactions between miRNAs and scrambled (randomly permuted) mRNA sequences. (B) Location of base-paired nucleotides in mRNAs. The mRNA fragments are ordered by the mean coordinate of the base-paired nucleotides in each fragment. Basepairing is usually confined to the first 25 nucleotides in CLASH targets. (C) Patterns of miRNA interactions in replicate experiments.

The large data set provided by AGO1-CLASH allowed the miRNA interactome to be characterized ab initio without utilizing prior knowledge of targets or binding modes. To re-evaluate the rules underlying miRNA interactions, we developed a graphical representation of miRNA-target RNA base pairing and applied K-means clustering to reveal five classes of interactions with distinct base-pairing patterns (Figures 3A and 3B and Data S2). Three of these classes (I–III) featured binding between the miRNA seed and the target but differed in the presence and positioning of additional base-paired nucleotides within the miRNA. Class I interactions are confined to the seed region, whereas classes II and III additionally involve miRNA nucleotides 13–16 and 17–21, respectively. In class IV, binding was limited to a region located in the middle and 3′ end of the miRNA, whereas class V showed distributed or less stable base pairing. The observed patterns of miRNA-mRNA interactions were largely absent among randomized pairs (Figures S3A and S3B). Evolutionary conservation and target downregulation were strongest in class II (Table 1), supporting the important role of 5′ and 3′ end base pairing in miRNA function (Broderick et al., 2011). The proportion of interactions that were supported by CLASH AGO1 single-read clusters or PAR-CLIP AGO1-4 binding clusters in mRNA was similar for each base pairing class (Figure 3C), suggesting that all classes are largely reliable.

**Table 1:** Analysis of the Five miRNA-mRNA Base-Pairing Classes

Class	I	II	III	IV	V
Number of interactions	3,594	3,293	4,630	3,389	3,608
Number of base-paired nucleotides	13.0 ± 0.04	15.3 ± 0.03	16.8 ± 0.03	14.6 ± 0.04	11.9 ± 0.05
Number of base-paired nucleotides in seed	5.2 ± 0.02	5.1 ± 0.02	5.0 ± 0.01	2.6 ± 0.02	3.3 ± 0.03
Interaction energy (dG)	−18.3 ± 0.04	−20.2 ± 0.06	−20.5 ± 0.05	−19.0 ± 0.05	−11.1 ± 0.05
PhyloP conservation score	0.092 ± 0.017	0.127 ± 0.018	0.097 ± 0.017	0.011 ± 0.017	0.086 ± 0.018
Efficiency of inhibition by miRNA	0.042 ± 0.007	0.052 ± 0.009	0.047 ± 0.005	0.024 ± 0.005	0.039 ± 0.004
Targets in 5′ UTR	4.8%	4.2%	4.1%	5.8%	4.7%
Targets in CDS	60.7%	61.1%	61.4%	63.9%	53.4%
Targets in 3′ UTR	32.7%	32.2%	32.1%	28.1%	39.5%

The number of predicted base pairs between the entire miRNA and the target or the miRNA seed region (nts 2–7) and the target was predicted using the RNAhybrid program from the UNAFold suite. The minimum free energy of interaction was calculated with RNAhybrid. The PhyloP conservation score was calculated as the difference between the average PhyloP score in the longest stem predicted in each interaction and the average PhyloP score in flanking genomic DNA (Pollard et al., 2010). The efficiency of target inhibition by miRNA was calculated as the average log2 fold enrichment of mRNA in miRNA-depleted versus control cells using published microarray data (Hafner et al., 2010a). The numbers in the table represent the mean with SE for each class of interactions. The proportion of targets in the 5′ UTR, CDS, and 3′ UTR was calculated using the annotations of ENST transcripts downloaded from Ensembl through Biomart. Overall, 60% of all targets were mapped to the coding sequence, and 35% were mapped to the 3′ UTR. The proportions of targets mapped to the 3′ UTRs are slightly lower compared to previous CLIP-seq experiments. We believe that this results from our method of mapping sequencing reads to a transcriptome database, which recovers reads mapped to splice junctions, thereby recovering more hits in coding sequences. When CLASH targets mapped to splice junctions are discarded, 50% of the remaining targets are mapped to the coding sequence, and 42% are mapped to the 3′ UTR.

Two-thirds of all miRNAs analyzed showed nonrandom distribution across the five base pairing classes. Most miRNAs, including let-7a, miR-10a, and miR-15b, were enriched in the seed-interacting classes I–III, but miR-92a and 11 other miRNAs showed highest enrichment in the nonseed class IV (Figure 3D). Comparison of the six different CLASH protocols indicated that protocol E4 yielded the largest proportion of chimeras in classes I–IV (Figure S3C).

### Analysis of Enriched Motifs on mRNA Targets Identifies the Major Interaction Site for Many miRNAs

**Figure 4:**
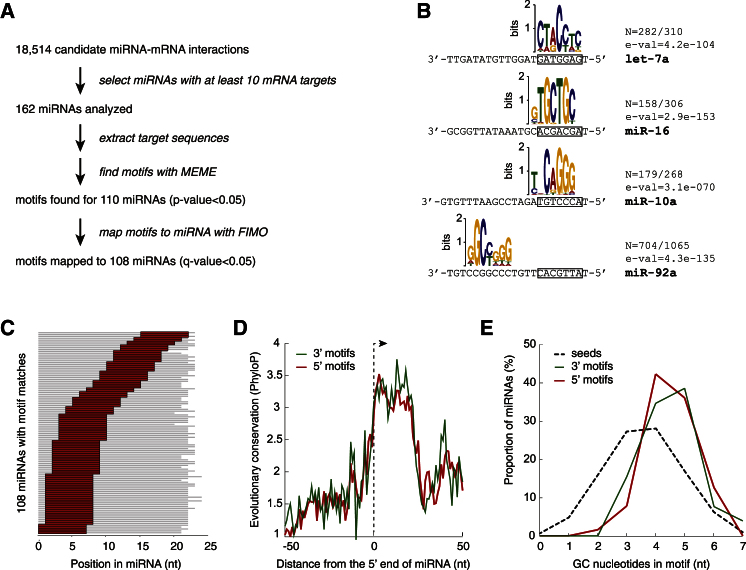
Sequence Motifs Associated with miRNA-Binding Sites (A) Discovery pipeline for overrepresented motifs in miRNA targets. Target sequences with 25 nt flanking genomic sequence were analyzed by MEME (Bailey and Elkan, 1994), and 7-mer motifs were considered. 108 could be mapped back to the miRNA by FIMO (Grant et al., 2011) with FDR < 0.05. (B) Example motifs bound by miRNA. n, number of motifs found/total number of targets analyzed. E-val, e-value of the motif returned by MEME. Most motifs are complementary to the miRNA seed (boxed). (C) Distribution of conserved motif positions within 108 miRNAs. In most cases, the motifs enriched in miRNA targets were complementary to the miRNA seed (nt 1–9); however, some highly enriched motifs were complementary to regions in the middle or 3′ ends of the miRNA. (D) Conservation patterns among 108 miRNAs with recognizable target motif sequences. miRNAs were partitioned by most enriched motif location into groups predicted to form seed and nonseed interactions. The 5′ half of the miRNA is more conserved among the seed-interacting group (average difference in PhyloP scores [Pollard et al., 2010] between 5′ and 3′ halves, Δ_PhyloP_ = 0.122, t test, p = 0.001). The 3′ half of the miRNA is more conserved among the nonseed interacting group (Δ_PhyloP_ = –0.164, p = 0.002). (E) Distribution of GC content in motifs (n = 108) and miRNA seeds (n = 1100). The average guanine plus cytosine (GC) content of the binding motifs was higher than the average GC content of miRNA seeds in human. See also Figure S4 and Table S5.

**Figure S4:**
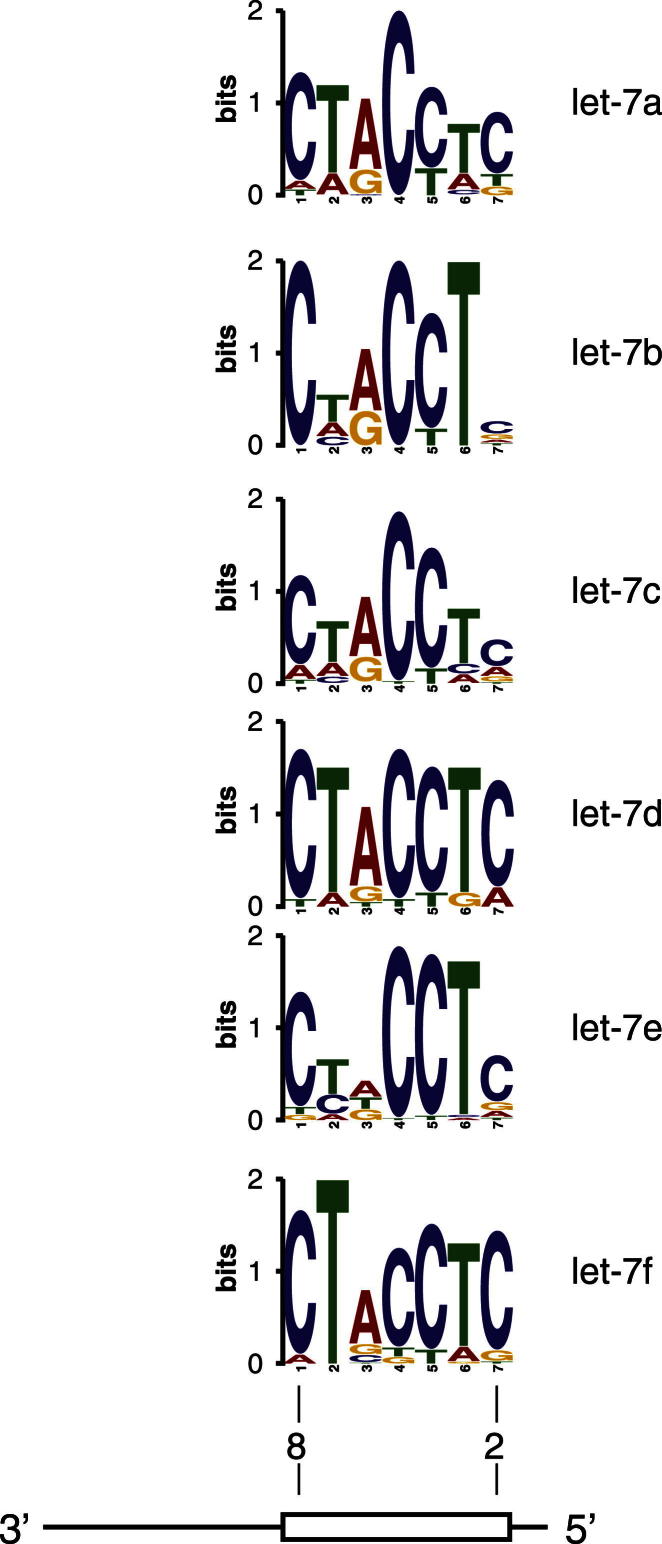
Comparison of Motifs Identified in Targets of let-7 Family miRNAs, Related to Figure 4

To identify additional features of miRNA-binding sites, we sought statistically overrepresented sequence elements in the CLASH targets of each miRNA using MEME (Bailey and Elkan, 1994) (Figure 4A). For many miRNAs, highly enriched sequence motifs emerged. In the majority of cases, the motifs were complementary to the miRNA seed region; however, motifs found for several miRNAs indicated preferred interactions with the 3′ region of the miRNA (Figures 4B and 4C and Table S5). Different miRNAs seem to follow idiosyncratic patterns of complementarity, but some common features emerge. For example, all six variants of let-7 yield almost exactly the same enriched motif that maps to nucleotides 2–8 of the miRNA (Figure S4). Interestingly, the nucleotide predicted to base pair with the U at position 6 in let-7 is the most variable. This pattern resembles the characterized *let-7::lin-41* interaction and the G bulges recently identified in target sequences located opposite positions 5–6 in the miRNA (Chi et al., 2012).

Many miRNAs are highly conserved in evolution; regions of highest conservation are typically the seed element (nt 1–8) and a downstream region (nt 13–19) (Grimson et al., 2007). We compared the sequence conservation patterns for miRNAs associated with 5′ (seed) or 3′ (nonseed) motifs (Figure 4D). Both in the case of 5′ and 3′ binding motifs, the part of the miRNA that contains the motif shows stronger evolutionary conservation than the part without motif (paired t test, p = 0.001 and 0.002 for 5′ and 3′ motifs, respectively). The identified motifs might be favored because they improve the efficiency of target RNA interactions with the AGO1-miRNA complex or because they influence the effects of miRNA binding on the fate of the mRNA. The identified motifs showed a higher average GC content than seed regions, suggesting selection for enhanced base pairing (Figure 4E).

### Experimental Validation of Noncanonical Interactions

**Figure 5:**
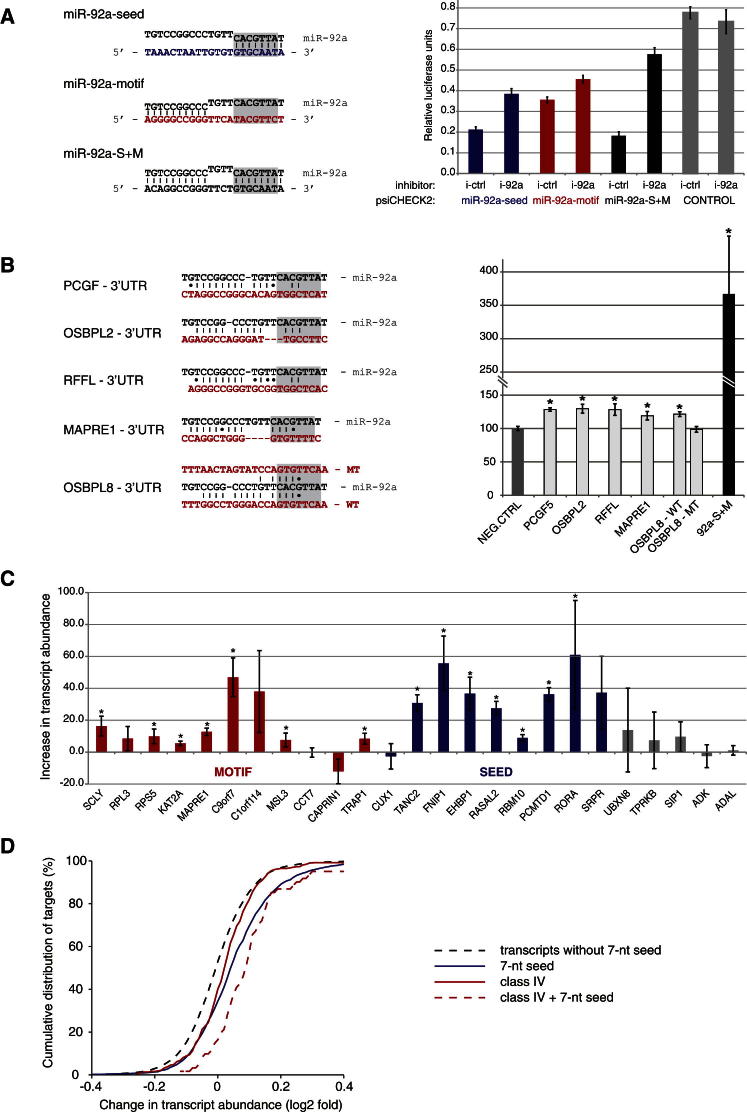
Experimental Validation of Noncanonical Interactions (A) Reporter vectors were constructed by inserting miR-92a-binding sites matching the seed, 3′ motif, or seed+3′ motif (S+M) (left) into the 3′ UTR of *Renilla* luciferase in a psiCHECK2 vector. *Renilla* to firefly luciferase ratios are shown with error bars representing SE from four independent experiments (right). All binding sites caused miR-92-dependent downregulation of luciferase expression (p < 0.05, t test). (B) Reporter vectors were constructed by inserting 3′ UTRs of identified class IV miR-92a targets into the 3′ UTR of *Renilla* luciferase in a psiCHECK2 vector. Mean changes in *Renilla* to firefly luciferase ratios upon treatment with miR-92a inhibitors are shown with error bars representing SE from at least three independent experiments (right). A schematic of the CLASH identified miR-92a-binding sites within those UTRs, and sites of mutagenesis within one of the reporters are depicted on the left. All wild-type-binding sites resulted in significant increase of Renilla luciferase signal (p < 0.05, t test, marked with an asterisk), and mutagenesis of identified binding site resulted in reverting this effect. (C) Experimental validation of selected CLASH targets with miR-92a seed-only binding sites (blue), miR-92a motif-only binding sites (red), or negative controls (gray). Increase in transcript abundance upon inhibition of endogenous miR-92a was quantified by qRT-PCR and internally normalized to GAPDH. The bars represent the average from three independent experiments, error bars represent SD, and samples with p < 0.05 (t test) are marked with an asterisk. (D) Changes in mRNA abundance upon miR-92a depletion in cells measured by microarrays. The graph shows a cumulative distribution of the log2 fold change (LFC) of mRNA abundance for various kinds of miR-92a targets. Transcripts without 7-mer seed serve as negative control. See also Figure S5.

To test the role of nonseed interactions in target regulation, we chose miR-92a that is abundant in HEK293 cells and shows clear preference toward 3′ end interactions (Figures 3D and 4B). We prepared reporter vectors by inserting potential target sequences into the 3′ UTR of *Renilla* luciferase (Figure 5A). Complementarity to only the seed region of miR-92a, to only the nonseed 3′ motif of miR-92a, or to both regions (S + M) each caused miR-92-dependent downregulation of luciferase expression. We have also analyzed five further reporters that included large fragments of 3′ UTRs of putative nonseed miR-92a targets. Four of these UTR regions contain no miR-92a seed matches (6 nt or longer), whereas one region contained a 7 nt seed match. All five reporters showed a statistically significant increase in expression on depletion of miR-92a (Figure 5B). This experiment shows that a nonseed interaction involving miR-92a can downregulate mRNA translation in the context of an entire 3′ UTR region.

To further test the ability of miR-92a to regulate various kinds of targets, miR-92a was depleted from HEK293 cells using specific inhibitors (Figure 5C), and the abundance of targets randomly selected from our data set matching either seed or nonseed 3′ motif was measured by qRT-PCR. miR-92a depletion resulted in increased abundance of 7/9 (78%) seed-matching targets and 7/11(63%) motif-matching targets.

**Figure S5:**
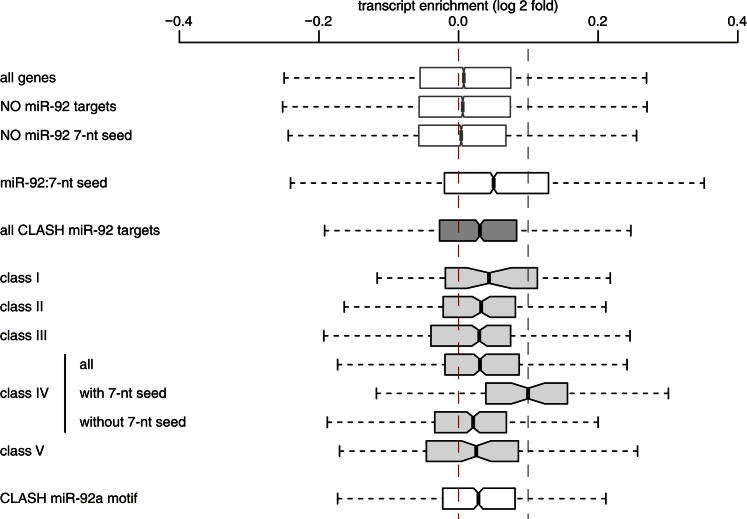
Experimental Validation of CLASH Identified miR-92a Targets, Related to Figure 5 Changes in mRNA abundance upon miR-92a depletion in PTH-AGO1-HEK293 cells, measured by Affymetrix microarrays. The performance of various classes of miR-92a targets identified in CLASH analyses, and targets containing the miR-92a motif, are compared to transcripts containing a match to the miR-92a 7-mer seed sequence (positive control), to random transcripts, and to targets lacking a match to the miR-92a 7-mer seed (negative control). The left and right edge of the box represent 25th and 75th percentile, respectively. The ends of the whiskers show the minimum and maximum values of the data.

We also quantified mRNA transcriptome wide using Affymetrix microarrays. We found that miR-92a targets with the 3′ motif (Figure 4B) were significantly upregulated after miR-92a depletion compared to genes not identified as miR-92a targets by CLASH (p < 1 × 10^−6^; Figures 5D and S5). Furthermore, mRNAs predicted to base pair with the 3′ end of miR-92a only (Figure 3B, cluster IV) are upregulated with respect to nontarget genes (p < 1 × 10^−5^). Although genes containing a miR-92a 7-mer seed match were upregulated relative to control, genes containing both a seed match and a cluster IV CLASH target were upregulated twice as highly (p = 0.003). Finally, genes containing a cluster IV CLASH target and no seed match were upregulated relative to genes containing neither a CLASH target nor a seed match (p = 0.007).

The CLASH data therefore identify a group of miRNAs that preferentially interact with their targets using nonseed regions. Nonseed interactions have statistically significant but only modest effects on mRNA stability and/or translation.

### miRNAs Target ncRNAs

**Figure 6:**
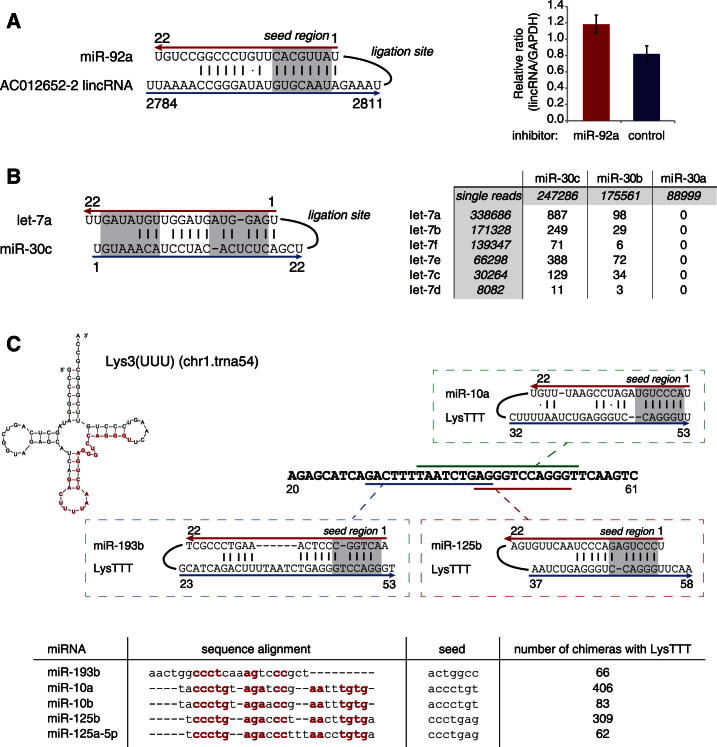
Examples of Interactions between miRNAs and Non-mRNA Targets (A) Experimentally validated, reproducible interaction between miR-92a and lincRNA AC012652-2 with canonical seed match. Change in the expression level of the lincRNA upon miR-92a inhibition was estimated by qRT-PCR. The error bar represents SE from three biological replicate experiments. (B) Putative interaction between miR-30 and let-7; left, folded structure of miR-30c- let-7a chimera; right, numbers of chimeras supporting the interactions between pairs of let-7 and miR-30 family members. The specificity of the interaction is supported by the presence of multiple chimeras between let-7 and miR-30b/c, and the absence of chimeras between let-7 and miR-30a. (C) Putative interactions between miRNAs and tRNA^Lys3(UUU)^. miR-10a, miR-10b, miR-125b, miR-125a-5p, and miR-193b bind with high reproducibility to the same region of tRNA^Lys3(UUU)^, marked red on the tRNA structure (chr1.trna54). As shown in the sequence alignment, these miRNAs have different seed sequences but are similar overall. See also Data S3.

AGO was previously shown to associate with a wide range of RNA species (Burroughs et al., 2011). We reproducibly recovered chimeras between a subset of miRNAs and other miRNAs, tRNAs, snRNAs, lincRNAs, and rRNAs (Figure 1D). As initial validation of non-mRNA interactions, we assessed the effects of miR-92a depletion on the lincRNA AC012652-2 (Figure 6A). Depletion of miR-92a resulted in upregulation of the lincRNA to an extent similar to validated mRNA targets, supporting their functional interaction. Notably, recent work by the Rajewsky and Kjems groups has identified a lncRNA (CDR1as) that acts as an endogenous sponge for miR-7 (Hansen et al., 2013; Memczak et al., 2013). Hybrids between miR-7 and the CDR1as transcript were identified in our analysis (data not shown) supporting the presence of this interaction in vivo.

miRNA-miRNA interactions were also reproducibly recovered. As an example, Figure 6B shows the interaction between members of the let-7 and miR-30 families. The six let-7 miRNAs recovered each interacted with miR-30c and miR-30b, but no let-7 chimeras were identified with miR-30a. Although somewhat fewer single reads were recovered for miR-30a than for miR-30b or miR-30c, the lack of chimeras indicates that the interactions are not random.

Chimeras between tRNA_Lys_^UUU^ and miR-10a/b, miR125a/b, and miR193b were each recovered in several independent experiments (Figure 6C). tRNA_Lys_^UUU^ is required as a primer for genome replication by reverse transcriptase for HIV-1 and other lentiviruses (Barat et al., 1989). The most numerous and highly reproducible non-mRNA chimeras were found with the 18S and 28S rRNAs. Different miRNAs showed very distinct patterns of rRNA interaction. Some miRNA-binding sites were located in exposed, surface regions and could have formed on intact, functional ribosomes, whereas other sites are internal to the ribosomal subunits and may reflect interactions with pre-ribosomes or degradation fragments. The interaction sites between miRNAs and all classes of non-protein-coding transcripts are listed in Data S3.

## Discussion

The aim of this study was to obtain an unbiased view of the human miRNA interactome and to use the information to re-evaluate the rules that govern miRNA-target base pairing. The 18,500 miRNA-mRNA interactions recovered provide a large data set of miRNA interactions that is independent of bioinformatic predictions. Multistep validation, which included structural, thermodynamic, evolutionary, and functional analysis, supports the reliability of our data. Moreover, a control CLASH experiment performed with mixed human and yeast lysates indicated that the large majority (>98%) of the miRNA-target RNAs interactions identified by CLASH had formed in vivo in human cells.

Although seed-mediated interactions constitute the largest class in our data, only around 37% of seed interactions involve uninterrupted Watson-Crick base pairing. This figure seemed surprisingly low but is consistent with the many observations of endogenous noncanonical miRNA targets. High-throughput studies found fewer noncanonical (or nonseed) interactions (Chi et al., 2009; Hafner et al., 2010b), but this may reflect an inherent bias in that seed binding was used to computationally identify interactions. Notably, many high-confidence AGO-binding sites identified in previous CLIP-seq data could not be assigned bioinformatically to any specific miRNA. Computational searches for miRNA-mRNA interactions have also been biased toward the identification of binding sites in 3′ UTR regions. In contrast, we observed substantial numbers of miRNA interactions in all the regions of mRNAs, with the greatest number of hits in coding sequences. Notably, different miRNAs vary in the relative proportions of targets in 5′ UTRs, coding sequences, and 3′ UTRs. As examples, miR-100 returned 4% 5′ UTR: 23% CDS: 73% 3′ UTR, whereas miR-149 returned 8% 5′ UTR: 72% CDS: 19% 3′ UTR (data not shown).

To provide an overview of the key features of miRNA-mRNA interactions, we analyzed miRNA base-pairing patterns by cluster analysis. As expected, the most frequent miRNA interaction site with a target is the seed, and base pairing in this region is detected for more than half of the interactions. However, seed interactions alone are found in only a relatively small fraction of identified targets (class I, 19%). Defined classes II–III agree with previously described 3′ supplementary and compensatory sites (Grimson et al., 2007; Lian et al., 2009). Unexpectedly, we identified a substantial class of interactions (class IV, 16% of all interactions) that does not involve contacts within the seed region and resembles reported “seedless” interactions (Lal et al., 2009). The identification of miRNAs that predominately interact with target mRNAs using their 3′ regions helps explain the pattern of evolutionary conservation of these miRNAs. However, target mRNAs that fall into this class seem to be relatively poorly conserved in evolution, and high-throughput data show that, on average, these targets respond only weakly to miRNA binding. Our experimental data on the regulation of miR-92a targets agree with this analysis, showing a statistically significant but moderate effect of class IV interactions on mRNA stability and possibly translation in reporter constructs. The results further suggested that the 3′ motif might act cooperatively with seed interactions. It is, of course, possible that the nonseed, motif interactions have additional functions, e.g., in attracting regulatory factors or switching effector pathways.

Overall, we show that noncanonical miRNA-mRNA targeting is much more widespread than anticipated. Moreover, the analysis of base-pairing patterns and of miRNA-binding site motifs indicates that individual miRNAs systematically differ in their target binding modes. Indeed, even members of the same miRNA family can manifest distinct base-pairing patterns. This was previously predicted by RepTar (Elefant et al., 2011) and was observed on a small scale in the analysis of enriched 6-mers in mRNAs recovered in AGO-immunoprecipitates following miRNA transfection (Nelson et al., 2011; Wang et al., 2010).

The recently published human AGO2 crystal structure (Elkayam et al., 2012) does not exclude the possibility of noncanonical seed interactions. The trajectory of the miRNA seen in the structure leaves most base edges accessible to be read by potential target molecules. Biochemical studies show that the structure of hAGO2 is flexible, and miRNA binding stabilizes and spatially orients AGO2 domains. Differences in patterns of miRNA-target RNA base pairing can induce allosteric changes in the RISC complex, potentially leading to different AGO activities. This suggests that the various interaction classes and/or the specific motifs identified might have distinct functional roles. The integration of CLASH data with RNA-sequencing and proteomics should give a clearer indication of the range of miRNA functions and their relationship to miRNA-mRNA interaction patterns.

Many interactions between Argonaute proteins and abundant, stable rRNA and tRNA species can be found in our data and in published high-throughput AGO-CLIP experiments (Chi et al., 2009; Hafner et al., 2010a). Evidence for miRNA-rRNA interactions has been reported, including the association of miR-206 with both nuclear preribosomes and mature cytoplasmic ribosomes (Politz et al., 2006). miR-206 is, however, specific for skeletal muscles and is not expressed in HEK293 cells. In addition, the involvement of AGO2 in pre-rRNA processing has been reported, although it is unclear whether this is dependent on the RISC pathway (Liang and Crooke, 2011). Specific, short tRNA fragments can be bound by AGO proteins and possibly function analogously to miRNAs (Burroughs et al., 2011), but there are no previous reports of tRNAs being targeted by miRNAs.

It was recently proposed that “competing endogenous RNA” (ceRNA), generated from transcribed pseudogenes and long noncoding RNAs, participates in mRNA regulation by competing for miRNA binding (Salmena et al., 2011). We speculate that regulation by competition for miRNAs involves not only ncRNAs and other modestly expressed species but also the abundant stable RNAs. In some cases, the highly abundant tRNAs and rRNAs may also “buffer” miRNAs. They might potentially bind miRNAs that are in (perhaps temporary) excess over cognate targets, preventing inappropriate target binding and/or protecting unbound miRNAs against premature degradation. This model is supported by the observation that miRNA interactions with mRNAs have a lower average free energy than those with stable RNA species (data not shown), so authentic target mRNAs might readily recruit cognate miRNAs from the buffered pool.

Interactions between pairs of distinct miRNAs were not very frequent (∼3%), but some were highly reproducible and apparently isoform specific—for example, miR-30::let-7. Two published reports of miRNA-miRNA interactions reveal different outcomes. Binding of miR-107 and let-7 mutually reduced miRNA stability and activity (Chen et al., 2011), whereas binding of miR-709 alters the biogenesis of miR-15a/16-1 (Tang et al., 2012).

The application of the CLASH technique to miRNAs offers many possibilities for future research. As an example, analyses of miRNA association reveal comparable distributions of miRNAs associated with the four mammalian AGO homologs (Burroughs et al., 2011; Liu et al., 2004; Meister et al., 2004; Su et al., 2009), but it is less clear whether all miRNAs target the same mRNAs when bound to different AGOs. Similarly, closely related paralogs exist for many human miRNAs, but it has been difficult to determine their relative efficiencies in mRNA targeting. The distribution of nontemplated terminal U residues among miRNAs has also been determined (Kim et al., 2010), but not how this effects targeting in vivo. More generally, the spectrum of miRNA-mRNA interactions is expected to rapidly change during differentiation, and viral infection and following metabolic shifts or environmental insults. All of these can potentially be addressed using CLASH.

## Experimental Procedures

### CLASH Analyses

The previously reported protocol (Kudla et al., 2011) was extensively modified to allow miRNA target identification in mammalian cells. The experimental protocol, variants tested, and bioinformatic analyses are described in detail in the Supplemental Information.

### Cell Lines

A protein A-TEV protease cleavage site 6xHis (PTH) tag was fused to the N terminus of human AGO1 and stably transfected into Flp-In T-REx 293 cells. PTH-AGO1 expression was induced with Doxycycline and confirmed by western blotting.

### Experimental Validation of CLASH Targets

Flp-In T-REx 293-hAGO1 cells were transfected with miR-92a inhibitor or universal negative control. 48 hr posttransfection RNA was isolated, and cDNA was quantified using primers listed in Table S6. Luciferase reporter vectors were prepared by cloning short oligonucleotides containing single miR-92a-binding sites or PCR-amplified long fragments of 3′ UTRs (sequences in Table S6) into the 3′ UTR of *Renilla* luciferase in the psiCHECK2 vector (Promega). HEK293 cells were transfected in 96-well plates with reporter vectors or nonmodified psiCHECK2 as control together with control or miR-92a inhibitors. Luminescence of *Renilla* and firefly (internal reference) luciferases was measured 48 hr posttransfection.

Extended Experimental Procedures **CLASH Protocol**

**Cell Preparation and Crosslinking**

Flp-In T-REx 293–PTH-AGO1 and control Flp-In T-REx 293 cells (Life Technologies) were seeded onto 150 mm plates (Nunc, Thermo Scientific), 4 plates per sample. The next day cells were induced for hAGO1 production with 0.5 μg/ml Doxycycline (Sigma, D9891). 36 hr post induction growing cells were UV crosslinked on ice with λ = 254 nm in Stratalinker 1800 (Stratagene), power settings = 400 mJ/cm^2^.

**Cell Lysis**

Directly after crosslinking cells were lysed by addition of cooled Lysis Buffer (50 mM Tris pH 7.8, 300 mM NaCl, 1% NP-40, 5 mM EDTA, 10% glycerol, 5 mM β-mercaptoethanol, protease inhibitors (Roche, cOmplete, EDTA-free)). Lysates were centrifuged at 6,400 x g for 30 min at 4°C and supernatant was collected. Unless used directly lysates were stored at −80°C.

**PTH-AGO1-RNA Purification on IgG-Dynabeads**

Dynabeads (Life Technologies, M-270 Epoxy) were coated in advance with rabbit IgG (Sigma, I5006) according to a published protocol (Oeffinger et al., 2007). Cell lysates were incubated with IgG-Dynabeads for 40 min at 4°C using 20 mg beads/sample and then beads were washed with LS-IgG WB buffer (50 mM Tris-HCl pH 7.8, 0.3 M NaCl, 5 mM MgCl_2_, 0.5% NP-40, 2.5% glycerol, 5 mM β-mercaptoethanol), HS-IgG WB buffer (50 mM Tris-HCl pH 7.8, 0.8 M NaCl, 10 mM MgCl_2_, 0.5% NP-40, 2.5% glycerol, 5 mM β-mercaptoethanol) and PNK-Wash buffer (50 mM Tris-HCl pH 7.5, 10 mM MgCl_2_, 0.5% NP-40, 50 mM NaCl, 5 mM β-mercaptoethanol).

**RNase A+T1 Digestion and PTH-AGO1-RNA Elution**

RNP complexes bound to IgG-Dynabeads were trimmed with 0.5 unit RNaseA+T1 mix (RNace-IT, Stratagene) in 500 μl PNK buffer (50 mM Tris-HCl pH 7.5, 10 mM MgCl_2_, 0.5% NP-40, 50 mM NaCl, 10 mM β-mercaptoethanol) for 7 min at 20°C. Then RNase solution was removed and PTH-AGO1-RNA complexes were eluted with Ni-WB I buffer (50 mM Tris-HCl pH 7.8, 300 mM NaCl, 10 mM Imidazole pH 8.0, 6 M Guanidine-HCl, 0.1 M NP-40, 5 mM β-mercaptoethanol) for 10 min at 20°C.

**Purification of PTH-AGO1-RNA on Ni-NTA Agarose**

The eluate from IgG-Dynabeads was loaded on 40 μl of Ni-NTA Agarose (QIAGEN) equilibrated with Ni-WB I for 2 hr at 4°C and then Ni-NTA beads were washed with Ni-WB I buffer. Ni-NTA beads were transferred to the spin columns (Pierce, Thermo Scientific, 69725) and from that point on all the reactions and washes were performed on the columns. Beads were subsequently washed with Ni-WB II buffer (50 mM Tris-HCl pH 7.8, 300 mM NaCl, 10 mM Imidazole pH 8.0, 0.1 M NP-40, 5 mM β-mercaptoethanol) and extensively washed with PNK-Wash buffer.

**RNA 5′ End Phosphorylation and Intramolecular Ligation**

Ni-NTA beads with bound PTH-AGO1-RNA complexes were incubated with 40 units T4 Polynucleotide kinase (New England Biolabs, M0201), 1 mM ATP and RNase inhibitors (RNasin, Promega, N211B) in PNK buffer for 150 min at 20°C, and then washed with Ni-WB I, Ni-WB II and PNK-Wash buffer. PTH-AGO1 bound, interacting RNA molecules were ligated together overnight using 40 units of T4 RNA ligase 1 (New England Biolabs, M0204), 1 mM ATP and RNase inhibitors in PNK buffer at 16°C. On the next day, the ligation mixture was washed out with Ni-WB I, Ni-WB II and PNK-Wash buffer.

**RNA Dephosphorylation and 3′ miRCat-33 Linker Ligation**

Ni-NTA beads were resuspended in a dephosphorylation mixture containing 8 units Thermosensitive Alkaline Phosphatase (Promega, M9910) and RNase inhibitors in PNK buffer for 45 min at 20°C and subsequently washed with Ni-WB I, Ni-WB II and PNK-Wash buffer. 3′ miRCat-33 linker ligation was performed for 6 hr at 16°C in the reaction mixture containing 800 units T4 RNA ligase 2 truncated, K227Q (New England Biolabs, M0351), 1 μM miRCat-33 linker (IDT), 10% PEG 8000 and RNase inhibitors in PNK buffer. Beads were washed with Ni-WB I, Ni-WB II and PNK-Wash buffer.

**Radioactive Labeling of RNA and Elution of PTH-AGO1-RNA Complexes**

RNAs bound to PTH-AGO1 were radiolabelled with ^32^P-γ-ATP (Perkin Elmer, 6000 Ci/mmol) in a mixture containing 40 units T4 Polynucleotide kinase, RNase inhibitors in PNK buffer for 30 min at 37°C. Beads were washed with Ni-WB I, Ni-WB II and PNK-Wash buffer. AGO1-RNA complexes were eluted by incubation with Ni-EB for 5 min at room temperature.

**TCA Precipitation, SDS-PAGE, and Transfer to Nitrocellulose**

Protein-RNA complexes from the Ni-NTA eluate were precipitated with 2 μg BSA (Sigma) and 17% TCA. Pellets were washed twice with cold acetone, dried, resuspended in 10 μl water and NuPAGE LDS SB (Life Technologies, NP0007). Samples were incubated for 10 min at 65°C and then resolved on a 4%–12% Bis-Tris NuPAGE gel (Life Technologies, NP0335) in NuPAGE SDS MOPS running buffer (Life Technologies, NP0001). Protein-RNA complexes were transferred to nitrocellulose membrane (GE Healthcare, Amersham Hybond ECL) in the wet-transfer tank (Bio-Rad, Mini Trans Blot cell) with NuPage transfer buffer (Life Technologies, NP00061) and 10% methanol for 2 hr at constant voltage = 100V, on ice. Air-dried membrane was exposed on film (GE Healthcare, Amersham Hyperfilm MP) for about 1 hr at room temperature. Developed film was aligned with the membrane and the radioactive bands corresponding to the PTH-hAGO1 complexes were cut out.

**Proteinase K Treatment and RNA Isolation**

Cut out bands were incubated with 100 μg of Proteinase K (Roche) and proteinase K buffer (50 mM Tris-HCl pH 7.8, 50 mM NaCl, 10 mM imidazole pH 8.0, 0.1% NP-40, 1% SDS, 5 mM EDTA, 5 mM β-mercaptoethanol) for 2 hr at 55°C. The membrane was discarded. Released RNA was extracted with phenol-chloroform-isoamyl alcohol (PCI) mixture and ethanol precipitated overnight with 20 μg GlycoBlue (Ambion, Life Technologies). Pellets were washed twice with 70% ice-cold EtOH, then air-dried.

**5′ Phosphorylation and 5′ Linker Ligation**

RNAs were phosphorylated with 10u of T4 Polynucleotide kinase in RNA ligase 1 buffer (New England Biolabs) for 30 min at 37°C. Then 10 units of RNA ligase 1 and barcoded 5′ linker (final conc. 5 μM; Table S6) were added and the reaction mixture was incubated for 10 hr at 16°C. RNA was subsequently PCI extracted end ethanol precipitated.

**Reverse Transcription and Library Amplification by PCR**

Whole isolated RNA was reverse transcribed with Superscript III Reverse Transcriptase (Life Technologies) according to manufacturer instructions, using miRCat-33 primer (IDT) at 50°C. RNA was then degraded by addition of 2 μl RNase H (New England Biolabs) for 30 min at 37°C. cDNA was amplified using primers P5 and primer PE_miRCat_PCR (Table S6) and TaKaRa LA Taq polymerase (Takara Bio, RR002M).

**Library Size Selection**

PCR products were separated on a 3% MetaPhor agarose (Lonza)/TBE gel with SYBRSafe (Life Technologies). The gel was run in 1 x TBE on ice. Then the gel was scanned on a Fujifilm scanner (Fla-5100) and two gel slices with different DNA fragments sizes were cut out: LB: 90 - 100 nt and HB: 110 - 180 nt. After purification with Gel Extraction Kit with MinElute columns (QIAGEN), libraries were stored at −20°C. For high-throughput sequencing, LB:HB fractions were mixed at a 1:3 ratio.

**Cell Lines**

Tagged human AGO1 was constructed by ligating the Protein A - TEV protease cleavage site - 6xHis (PTH) tag (amplified from the pRS415 plasmid, a generous gift from Markus T. Bohnsack, Frankfurt, Germany) to the N-terminus of human AGO1 amplified from cDNA, which was then cloned into the pcDNA5/FRT/TO vector (Life Technologies). This vector was further used for stable transfection of Flp-In T-REx 293 cells (Life Technologies) according to the manufacturer’s protocol. Before the CLASH experiments PTH-AGO1 expression was induced with Doxycycline (final concentration = 0.5 μg/ml) for 36 hr, with the aim of achieving expression close to that of endogenous AGO1. Expression of tagged AGO1 was confirmed by the Western Blotting, using peroxidase anti-peroxidase soluble complex (PAP; Sigma, P1291) recognizing the protein A tag.

**Determination of the Background Level of RNA-RNA Interactions**

To assess the frequency at which RNA-RNA interactions recovered by CLASH were formed in vitro following cell lysis, lysate obtained from the crosslinked HEK cells expressing PTH-AGO1 was mixed with an equal quantity (measured by RNA content) of yeast cell lysate, prior to CLASH analysis based on the optimized protocol (E4). Analysis of a sample in which no yeast lysate was present (E7) showed the background of human sequences that could be, incorrectly, matched to the yeast genome (0.37% of single reads and 0.11% of miRNA chimeras) (Table S4). Correcting for this, experiment E8, in which yeast lysate was included, reveals that 1.1% of single reads and 1.6% miRNA chimeras arose from yeast RNAs. This low level of background probably reflects the very stringent purification conditions used during CLASH. The same analysis was performed for data sets E9 and E10, prepared using an alternative protocol (E6), in which ligation was performed prior to protein denaturation. This gave a higher level of yeast-human chimeras (∼10%) (Table S4). This indicates that variations in the CLASH protocol can have substantial effects on the signal to noise ratio in the resulting cDNA library, and also confirms the conclusion that protocol E4 is optimal.

**Experimental Validation of CLASH Targets by qPCR**

Flp-In T-REx 293-hAGO1 cells were transfected with 25nM miR-92a inhibitor or universal negative control (miRIDIAN Hairpin inhibitors, ThermoScientific, IH-300510-06, IN-001005-01) and Lipofectamine 2000 (Life Technologies). 48 hr post-transfection RNA was isolated using TRI Reagent (Sigma, T9424), DNase treated (TURBO DNase, Ambion) and further purified by ethanol precipitation. cDNA was prepared using SuperScript III (Life Technologies) and random 6-mer oligonucleotides (Promega) at 45°C. cDNA was quantified using Roche LightCycler 480 Real-Time PCR System, Universal Probe Library System (Roche) and primers listed in Table S6. All the primers were designed using Roche UPL Assay Design Center. The expression level of the genes of interest (GIO) was internally normalized to the expression level of GAPDH.

**Luciferase Reporter Assays**

Luciferase reporter vectors were prepared by cloning short oligonucleotides containing single miR-92a-binding sites or PCR amplified long fragments of target 3′UTRs into the 3′ UTR of *Renilla* luciferase in the psiCHECK2 vector (Promega) using XhoI and NotI restriction sites. All the sequences of oligonucleotides used for creating reporters and reporter mutagenesis are listed in the Extended Experimental Procedures. HEK293 cells were transfected in 96-well plates with 50 or 10ng of reporter vectors or non-modified psiCHECK2 as control together with 25nM control or miR-92a inhibitors (miRIDIAN Hairpin inhibitors, Dharmacon, IN-001005-01 and IH-300510-06) or 6.25nM miR-92a controls and inhibitors (IDT, custom oligonucleotides, sequences listed in Extended Experimental Procedures) using Lipofectamine 2000 (Life Technologies). Luminescence of *Renilla* and firefly (internal reference) luciferases was measured 48 hr post-transfection using the Dual-Glo Luciferase Assay System (Promega, E2920) according to manufacturers’ instructions and SpectraMax M5 Multi-Mode Microplate Reader (Molecular Devices).

**Microarray Analysis**

Flp-In T-REx 293-hAGO1 cells were transfected with 6.25nM miR-92a inhibitor or negative control (IDT synthesized custom oligos, sequences in the list of oligonucleotides) and Lipofectamine 2000 (Life Technologies), each sample in 5 replicates. 48 hr post-transfection RNA was isolated using TRIZOL (Invitrogen) and concentrated by ethanol precipitation. Total RNA was processed and quantified on Affymetrix GeneChip Human Exon 1.0 ST by Source BioScience.

**Bioinformatic Analysis**

**BLAST Transcriptome Database**

The components of our custom BLAST database come from the following sources:

(1) BioMart (http://www.biomart.org), data set: GRCh37.p2: mRNA (cDNA sequence of protein coding genes limited to those with RefSeq protein ID), pseudogenes (miRNA_pseudogene, misc_RNA_pseudogene, Mt_tRNA_pseudogene, polymorphic_pseudogene, RNA_pseudogene, snoRNA_pseudogene, snRNA_pseudogene, tRNA_pseudogene), snoRNA, snRNA, processed transcripts, lincRNA, miscellaneous RNA, mitochondrial rRNA, mitochondrial tRNA, 5.8S rRNA, 5S rRNA;
(2) genomic tRNA database http://gtrnadb.ucsc.edu/): human tRNAs;
(3) National Center for Biotechnology Information (http://www.ncbi.nlm.nih.gov): rRNA sequence (NR_003287.2, NR_003286.2);
(4) miRBase release 15 (http://www.mirbase.org): mature human miRNAs.

Redundant sequences were removed from the database, as were pseudogenes, lincRNAs and processed transcripts that matched full-length mature miRNA sequences. The mRNA sequences and coordinates in the database match those of ENST entries from ENSEMBL Release 60 (http://useast.ensembl.org/info/website/archives/index.html). For the analysis of experiments E7-E10 we supplemented our database with sequences of *S. cerevisiae* chromosomes obtained from the *Saccharomyces* Genome Database.

**Mapping Reads by BLAST**

Illumina reads were assigned to the experimental and control samples using their 5′ barcode sequences, and barcodes were clipped. Illumina reads were then mapped by nucleotide BLAST (Altschul et al., 1990) version 2.2.24 to the human transcriptome database described above. BLAST was run with the expectation value set to 0.1, and other parameters set to default. We discarded all antisense matches. Approximately 70% of all mapped reads were mapped unambiguously. The remaining sequences mapped to more than one transcript, typically originating from a single gene. To uniquely assign those reads that were mapped with the same e-value to more than one transcript, we ranked all transcripts according to their total number of BLAST hits and assigned the read to the transcript with most hits.

**Identification and Clustering of Chimeras**

We identified as chimeras those reads where: (1) the read yielded at least two blast hits with e-value ≤ 0.1 against the human transcriptome database described above; (2) the hits were either directly adjacent in the read, or with up to 4 nucleotides gap or overlap between hits; and (3) the hits were in sense orientation with respect to the blast database. We rejected reads with more than ten hits in the database. For reads that could be assigned to chimeras in more than one way (for example if one of the fragments was mapped to two different transcripts from the same gene), we used the following criteria to decide which mapping to retain, in the following order: (1) we retained the mapping with the most significant e-value of both fragments; (2) we preferentially retained miRNA-mRNA chimeras; (3) we retained the mapping to transcripts with the highest number of non-chimeric reads. Criterion (2) was introduced to avoid assigning chimeras to pseudogenes, if an mRNA transcript with the same mapping quality was found.

After mapping the chimeric reads, we clustered those chimeras for which the coordinates of both mapped fragments overlapped, independently of the order of fragments in the chimera. We first performed the clustering for each of the six experiments independently, and then clustered the six experiments together. Finally we adjusted the transcript regions found in clustered chimeras by extending the miRNA fragment to the full length mature miRNA, and by adding 25 nucleotides downstream sequence to each mRNA fragment. We refer to the mRNA fragments adjusted in this way as the “CLASH targets,” and to the miRNA-mRNA fragment pairs as “CLASH interactions.”

**Identification of AGO1-Binding Site Clusters from Nonchimeric Reads**

We identified clusters of non-chimeric reads using a method similar to that previously described (Chi et al., 2009). First, we randomly distributed all distinct reads mapped to each transcript along the same transcript using BEDTools (Quinlan and Hall, 2010), to calculate the maximum random cluster height for each transcript. We then repeated the procedure a hundred times, and retained the maximum cluster height across the repeats. This value was used as a transcript-specific cluster height threshold for the identification of actual clusters. High-confidence clusters were identified as regions of transcripts in which the coverage equaled or exceeded the threshold, the coverage was higher than twenty reads, and the region of high coverage was at least twenty-nucleotide wide. We then calculated high-confidence cluster peaks as the area of twenty nucleotides on each side of the position of highest coverage within the cluster.

**Overlap between CLASH Targets and Experimentally Validated miRNA Targets from Published Databases**

To check if our CLASH data set contains known, experimentally validated miRNA targets we compared it with two databases – miRTarBase (Hsu et al., 2011) and TarBase (Vergoulis et al., 2012). Information in these databases is limited: target and miRNA names are often ambiguous (ex. LIN-28B and Lin28 are both used to identify the same gene), and detailed information about the binding site is often missing. To make the comparison possible we downloaded both databases and modified them as follows: 1) we simplified all the miRNA names to “miR-number” (and let-7); 2) we filtered TarBase to only retain human genes with an ENSEMBL gene ID (ENSG) represented in our custom database; 3) we translated gene names in miRTarBase to ENSEMBL gene IDs using the “Gene ID Conversion” tool from Database for Annotation, Visualization and Integrated Discovery (DAVID) (Dennis et al., 2003) and only genes represented in our custom database were retained; 4) we removed redundant interactions within each database. As a consequence each miRNA-mRNA interaction was represented in a simple “miRNA – ENSG” fashion.

**Overlap between CLASH Targets, AGO-Binding Sites, and Bioinformatically Predicted miRNA Targets**

The number of CLASH targets that overlapped with AGO1-binding sites obtained in the present study was estimated using BEDTools (Quinlan and Hall, 2010) intersect, using either the positions of all non-chimeric reads mapped to RNA, or the positions of high-confidence cluster peaks calculated as described above.

To calculate the overlap between CLASH targets and high-confidence AGO-binding sites obtained in the PAR-CLIP study we first mapped the sequences of the 17,319 clusters from Table S4 in (Hafner et al., 2010a) to our transcriptome database. 15823 clusters were successfully mapped. We then used BEDTools to intersect the positions of CLASH targets and AGO binding clusters.

To calculate the degree of overlap between CLASH targets and AGO-binding clusters that would be expected by chance, we randomly placed AGO-binding clusters on: (1) the same transcript in which each cluster was found; (2) a set of random transcripts with a distribution of expression levels matching the transcripts with AGO clusters; or (3) a set of transcripts randomly selected from the human transcriptome. The expression levels of human transcripts in 293 cells were obtained from the microarray data in (Hafner et al., 2010a). We then calculated the number of CLASH targets that overlapped the randomly placed clusters using BEDTools.

To calculate the overlap between CLASH targets and bioinformatically predicted miRNA targets by miRanda (John et al., 2004), TargetScan (Lewis et al., 2005), PITA (Kertesz et al., 2007), PicTar (Krek et al., 2005) and RNAhybrid (Rehmsmeier et al., 2004), we extracted the coordinates of predicted miRNA target sites in the hg18 genome from the Functional RNA Project website (www.ncrna.org). We converted CLASH targets from transcriptome to hg19 coordinates using a custom script based on the ENSEMBL Perl API (available on demand), then to hg18 coordinates using liftOver (Kent et al., 2002), and retained the targets mapped to a single genomic position that corresponded to a 3′UTR of a RefSeq gene, only considering those 3′UTR fragments that did not overlap with a CDS of a RefSeq gene on either strand. To generate the control interaction data set, we randomly distributed CLASH targets in the 3′UTR of RefSeq genes (excluding those fragments overlapping with a CDS). We then used BEDTools to overlap CLASH or control interactions with bioinformatically predicted targets. The enrichment was calculated as N_CLASH/N_control, where N_CLASH is the number of CLASH interactions that matched bioinformatic predictions, and N_control is the number of control interactions that matched bioinformatic predictions. To call a match we required that the CLASH interaction and the prediction share both (1) the miRNA name (2) the target position in the mRNA. To decide whether the CLASH targets and predictions refer to the same or different miRNA, we converted miRNA names to a simplified miR-number format, using the regular expression /[a-zA-Z]^∗^-[0-9]^∗^/.

**Analysis of Base Pairing in miRNA-Target Interactions**

To calculate free energies of binding and determine base pairing between miRNA and target fragments of chimeric reads we used programs from the UNAFold suite (Markham and Zuker, 2008). First, chimeric reads were divided into miRNA and target fragments based on the BLAST analysis. If the miRNA sequence was trimmed then it was replaced with the full-length miRNA sequence from miRBase (Griffiths-Jones et al., 2008). Target fragments were extended by 25 nucleotides at the 3′ end in case the length of the sequencing reads (which ranged from 50 to 100 nts) was too short to include the miRNA-binding site. Then we used the hybrid-min program with default parameters to calculate minimum hybridization energy of the two fragments of chimera that represented interacting RNA molecules. To convert the numeric representation into a Vienna-style dot-bracket format we used a modified version of M. Zuker’s Ct2b.pl script.

**Evolutionary Conservation of CLASH Targets**

To analyze evolutionary conservation of CLASH targets, we used per-nucleotide conservation scores among 46 vertebrate genomes calculated using the PhyloP algorithm (Pollard et al., 2010), and downloaded from the UCSC genome browser (Fujita et al., 2011). We selected CLASH targets that were located in 3′UTRs of RefSeq genes, with at least 100 nt of 3′UTR sequence on each side. The CLASH targets were centered at the 5′ end of the longest predicted miRNA-mRNA stem in each target. To calculate the average profile of evolutionary conservation around CLASH targets, the PhyloP score was calculated in the region from −100 to +100 nt from the center, averaged across all targets, and normalized to the number of targets for which the PhyloP score was available.

The conservation scores for individual CLASH targets were defined as (C_CLASH - C_control), where C_CLASH is the mean PhyloP score within the longest predicted miRNA-mRNA stem in each target, and C_control is the mean PhyloP score in regions spanning nt −50 through −10 and +10 through +50, relative to the ends of the stem. We used a paired t test to compare the mean conservation within and outside the predicted stems.

**Analysis of CLASH Target Downregulation after Inhibition of 25 miRNAs**

To analyze the effect of miRNA inhibition on mRNA transcript levels in specific sets of transcripts, we used the miRNA depletion data from (Hafner et al., 2010a). In this study, 25 miRNAs were depleted using an antisense 2′-O-methyl oligonucleotide cocktail, and mRNA levels were measured by the Human Genome U133 Plus 2.0 Array from Affymetrix. We averaged the log2 GC-RMA signal for all probes matching each transcript, discarding probes matching multiple genes. Probes were assigned to transcripts using data downloaded from ENSEMBL. We then calculated the log2-enrichment of each transcript upon miRNA depletion, by subtracting the average signal in mock-transfected cells from the average signal in 2′-O-methyl oligonucleotide-transfected cells.

To calculate the background distribution of mRNA enrichment upon depletion of 25 miRNAs, we randomly selected one transcript for each gene, and then selected a subset of transcripts with expression levels matching those of transcripts in which CLASH targets were identified. We plotted the cumulative distribution of log2-enrichment for these transcripts. As a positive control we used the 331 experimentally validated targets of the 25 miRNAs for which depletion data was available, downloaded from the miRTarBase on August 1, 2011.

We then plotted cumulative distributions of log2-enrichment scores after miRNA depletion, for the 1,995 transcripts in which CLASH targets for at least one of the 25 miRNAs were found, or for subsets filtered by: seed complementarity; location of the CLASH target in 5′ UTR, coding sequence, or 3′ UTR; predicted basepairing energy between miRNA and its target; presence of non-chimeric read clusters overlapping with the mRNA fragment of each chimera.

**Analysis of CLASH Target Downregulation after miR-92a Inhibition**

Data acquisition and normalization from Affymetrix GeneChip Human Exon 1.0 ST arrays were done by Source BioScience. We averaged the log2 RMA signal for all probes matching each transcript, discarding probes matching multiple genes. We only accepted probes for which expression was detected at p < 0.05 in at least 4 experiments. Probes were assigned to transcripts using data downloaded from ENSEMBL. We then calculated the log2-enrichment of each transcript upon miRNA depletion, by subtracting the average signal in mock-transfected cells from the average signal in miR-92a inhibitor-transfected cells. Wilcoxon tests were used to check whether the log2-enrichment differs between sets of genes.

**Identification of Base-Pairing Classes by K-Means Clustering**

We extracted the miRNA basepairing pattern for each CLASH interaction using a modified version of the Ct2B.pl script written by M. Zuker. We then converted the dot and bracket strings into a numeric format by replacing dots with zeroes, and brackets with a number F that represented the minimum energy of each interaction, rescaled from 0 to 5. This was calculated as F = 5^∗^(dG-dG_low_)/(dG_high_-dG_low_), where dG is the minimum energy of the interaction as calculated by hybrid-min, dG_low_ was set to −11 kcal/mol, and dG_high_ to −16 kcal/mol. F was capped at 0 at the bottom and 5 at the top.

We next performed K-means clustering on the matrix of 18,514 interactions and 22 miRNA positions using Gene Cluster 3.0 (downloaded from http://bonsai.hgc.jp/∼mdehoon/software/cluster/software.htm), to group interactions with similar basepairing patterns. We performed 50 runs of K-means clustering with K set to 4, 5, or 6, using Euclidean distance, and we selected the grouping into 5 clusters for presentation, as it yielded a clear separation into well-defined classes of comparable size.

We used two independent approaches to analyze the clustering patterns expected by chance. First, we randomly reassigned (shuffled) miRNA-mRNA pairs found in chimeras, before performing the folding prediction and clustering. This overestimated the number of interactions expected among random miRNA and mRNA fragments because many targets of abundant miRNAs were assigned by chance to the same miRNA. As a second approach, we randomly permuted (scrambled) the target sequence found in each interaction, while conserving the nucleotide content of the targets. Again, this approach is conservative because keeping the nucleotide content will act to increase apparent interaction strengths. Nevertheless, the basepairing patterns observed in the two randomized sets were very different from the one seen for CLASH interactions.

The basepairing patterns were represented graphically as heatmaps using Java TreeView (Saldanha, 2004), using contrast = 5.0 and a linear color scale. To analyze basepairing patterns for subsets of CLASH interactions, the clustering was performed first for the entire data set and the relevant interactions were extracted next, to preserve the order of clusters. Clustering each subset independently yielded similar results.

**Identification of Enriched Sequence Motifs in CLASH Targets**

To find overrepresented motifs in CLASH targets, we first extracted all miRNAs with at least 10 targets found. For each miRNA separately we ran MEME (Bailey and Elkan, 1994), with the settings: -dna -mod zoops -maxw 7 -nmotifs 1, using the default 0-order Markov model based on nucleotide frequencies in the training set as the background model. Changing the maximum motif length to 8 or 9; the maximum number of motifs to 2, or the number of nucleotides by which CLASH targets were bioinformatically extended between 0 and 50 did not change the conclusions.

The motifs identified by meme for targets of each miRNA were then aligned to the reverse-complemented miRNA sequence using FIMO (Grant et al., 2011), with the setting–output-pthresh 0.01. We then selected the motifs with FIMO q-value (FDR) < 0.05, and meme Bonferroni-corrected p-value < 0.05, yielding a set of 108 high-confidence motifs. For motifs that could be mapped to the reverse-complemented miRNA sequence in more than one way, the mapping with the most significant q-value was selected. (Details of the MEME analysis available upon request).

## Accession Numbers

The GEO accession number for the microarray data reported in this paper is GSE46039.

## Supplemental Information

Data S1. Detailed Annotation of All Identified miRNA-mRNA Interactions, Related to Figure 2

Data S2. Base Pairing between miRNAs and Their mRNA Targets, Related to Figure 3

Data S3. Base Pairing between miRNAs and Their Non-mRNA Targets, Related to Figure 6

Document S1. Tables S1–S6

Document S2. Article plus Supplemental Information

## References

[bib1] Altschul S.F., Gish W., Miller W., Myers E.W., Lipman D.J. (1990). Basic local alignment search tool. J. Mol. Biol..

[bib2] Bailey, T.L., and Elkan, C. (1994). Fitting a mixture model by expectation maximization to discover motifs in biopolymers. Proceedings of the Second International Conference on Intelligent Systems for Molecular Biology, 28–36. 7584402

[bib3] Barat C., Lullien V., Schatz O., Keith G., Nugeyre M.T., Grüninger-Leitch F., Barré-Sinoussi F., LeGrice S.F., Darlix J.L. (1989). HIV-1 reverse transcriptase specifically interacts with the anticodon domain of its cognate primer tRNA. EMBO J..

[bib4] Bartel D.P. (2009). MicroRNAs: target recognition and regulatory functions. Cell.

[bib5] Broderick J.A., Salomon W.E., Ryder S.P., Aronin N., Zamore P.D. (2011). Argonaute protein identity and pairing geometry determine cooperativity in mammalian RNA silencing. RNA.

[bib6] Burroughs A.M., Ando Y., de Hoon M.J., Tomaru Y., Suzuki H., Hayashizaki Y., Daub C.O. (2011). Deep-sequencing of human Argonaute-associated small RNAs provides insight into miRNA sorting and reveals Argonaute association with RNA fragments of diverse origin. RNA Biol..

[bib7] Chatterjee S., Grosshans H. (2009). Active turnover modulates mature microRNA activity in Caenorhabditis elegans. Nature.

[bib8] Chen P.S., Su J.L., Cha S.T., Tarn W.Y., Wang M.Y., Hsu H.C., Lin M.T., Chu C.Y., Hua K.T., Chen C.N. (2011). miR-107 promotes tumor progression by targeting the let-7 microRNA in mice and humans. J. Clin. Invest..

[bib9] Chi S.W., Zang J.B., Mele A., Darnell R.B. (2009). Argonaute HITS-CLIP decodes microRNA-mRNA interaction maps. Nature.

[bib10] Chi S.W., Hannon G.J., Darnell R.B. (2012). An alternative mode of microRNA target recognition. Nat. Struct. Mol. Biol..

[bib11] Elefant N., Altuvia Y., Margalit H. (2011). A wide repertoire of miRNA binding sites: prediction and functional implications. Bioinformatics.

[bib12] Elkayam E., Kuhn C.D., Tocilj A., Haase A.D., Greene E.M., Hannon G.J., Joshua-Tor L. (2012). The structure of human argonaute-2 in complex with miR-20a. Cell.

[bib13] Fabian M.R., Sonenberg N., Filipowicz W. (2010). Regulation of mRNA translation and stability by microRNAs. Annu. Rev. Biochem..

[bib14] Garcia D.M., Baek D., Shin C., Bell G.W., Grimson A., Bartel D.P. (2011). Weak seed-pairing stability and high target-site abundance decrease the proficiency of lsy-6 and other microRNAs. Nat. Struct. Mol. Biol..

[bib15] Grant C.E., Bailey T.L., Noble W.S. (2011). FIMO: scanning for occurrences of a given motif. Bioinformatics.

[bib16] Grey F., Tirabassi R., Meyers H., Wu G., McWeeney S., Hook L., Nelson J.A. (2010). A viral microRNA down-regulates multiple cell cycle genes through mRNA 5’UTRs. PLoS Pathog..

[bib17] Grimson A., Farh K.K., Johnston W.K., Garrett-Engele P., Lim L.P., Bartel D.P. (2007). MicroRNA targeting specificity in mammals: determinants beyond seed pairing. Mol. Cell.

[bib18] Ha I., Wightman B., Ruvkun G. (1996). A bulged lin-4/lin-14 RNA duplex is sufficient for Caenorhabditis elegans lin-14 temporal gradient formation. Genes Dev..

[bib19] Hafner M., Landthaler M., Burger L., Khorshid M., Hausser J., Berninger P., Rothballer A., Ascano M., Jungkamp A.C., Munschauer M. (2010). Transcriptome-wide identification of RNA-binding protein and microRNA target sites by PAR-CLIP. Cell.

[bib20] Hafner M., Landthaler M., Burger L., Khorshid M., Hausser J., Berninger P., Rothballer A., Ascano M., Jungkamp A.C., Munschauer M. (2010). PAR-CliP—a method to identify transcriptome-wide the binding sites of RNA binding proteins. J. Vis. Exp..

[bib21] Hansen T.B., Jensen T.I., Clausen B.H., Bramsen J.B., Finsen B., Damgaard C.K., Kjems J. (2013). Natural RNA circles function as efficient microRNA sponges. Nature.

[bib22] Hsu S.D., Lin F.M., Wu W.Y., Liang C., Huang W.C., Chan W.L., Tsai W.T., Chen G.Z., Lee C.J., Chiu C.M. (2011). miRTarBase: a database curates experimentally validated microRNA-target interactions. Nucleic Acids Res..

[bib23] John B., Enright A.J., Aravin A., Tuschl T., Sander C., Marks D.S. (2004). Human microRNA targets. PLoS Biol..

[bib24] Kertesz M., Iovino N., Unnerstall U., Gaul U., Segal E. (2007). The role of site accessibility in microRNA target recognition. Nat. Genet..

[bib25] Kim Y.K., Heo I., Kim V.N. (2010). Modifications of small RNAs and their associated proteins. Cell.

[bib26] Krek A., Grün D., Poy M.N., Wolf R., Rosenberg L., Epstein E.J., MacMenamin P., da Piedade I., Gunsalus K.C., Stoffel M., Rajewsky N. (2005). Combinatorial microRNA target predictions. Nat. Genet..

[bib27] Kudla G., Granneman S., Hahn D., Beggs J., Tollervey D. (2011). Cross-linking, ligation, and sequencing of hybrids reveals RNA-RNA interactions in yeast. Proc. Natl. Acad. Sci. USA.

[bib28] Lal A., Navarro F., Maher C.A., Maliszewski L.E., Yan N., O’Day E., Chowdhury D., Dykxhoorn D.M., Tsai P., Hofmann O. (2009). miR-24 Inhibits cell proliferation by targeting E2F2, MYC, and other cell-cycle genes via binding to “seedless” 3’UTR microRNA recognition elements. Mol. Cell.

[bib29] Lewis B.P., Burge C.B., Bartel D.P. (2005). Conserved seed pairing, often flanked by adenosines, indicates that thousands of human genes are microRNA targets. Cell.

[bib30] Li Y., Zhang M., Chen H., Dong Z., Ganapathy V., Thangaraju M., Huang S. (2010). Ratio of miR-196s to HOXC8 messenger RNA correlates with breast cancer cell migration and metastasis. Cancer Res..

[bib31] Lian S.L., Li S., Abadal G.X., Pauley B.A., Fritzler M.J., Chan E.K. (2009). The C-terminal half of human Ago2 binds to multiple GW-rich regions of GW182 and requires GW182 to mediate silencing. RNA.

[bib32] Liang X.H., Crooke S.T. (2011). Depletion of key protein components of the RISC pathway impairs pre-ribosomal RNA processing. Nucleic Acids Res..

[bib33] Liu J., Carmell M.A., Rivas F.V., Marsden C.G., Thomson J.M., Song J.J., Hammond S.M., Joshua-Tor L., Hannon G.J. (2004). Argonaute2 is the catalytic engine of mammalian RNAi. Science.

[bib34] Markham N.R., Zuker M. (2008). UNAFold: software for nucleic acid folding and hybridization. Methods Mol. Biol..

[bib35] Meister G., Landthaler M., Patkaniowska A., Dorsett Y., Teng G., Tuschl T. (2004). Human Argonaute2 mediates RNA cleavage targeted by miRNAs and siRNAs. Mol. Cell.

[bib36] Memczak S., Jens M., Elefsinioti A., Torti F., Krueger J., Rybak A., Maier L., Mackowiak S.D., Gregersen L.H., Munschauer M. (2013). Circular RNAs are a large class of animal RNAs with regulatory potency. Nature.

[bib37] Nelson P.T., Wang W.X., Mao G., Wilfred B.R., Xie K., Jennings M.H., Gao Z., Wang X. (2011). Specific sequence determinants of miR-15/107 microRNA gene group targets. Nucleic Acids Res..

[bib38] Poliseno L., Salmena L., Zhang J., Carver B., Haveman W.J., Pandolfi P.P. (2010). A coding-independent function of gene and pseudogene mRNAs regulates tumour biology. Nature.

[bib39] Politz J.C., Zhang F., Pederson T. (2006). MicroRNA-206 colocalizes with ribosome-rich regions in both the nucleolus and cytoplasm of rat myogenic cells. Proc. Natl. Acad. Sci. USA.

[bib40] Pollard K.S., Hubisz M.J., Rosenbloom K.R., Siepel A. (2010). Detection of nonneutral substitution rates on mammalian phylogenies. Genome Res..

[bib41] Reczko M., Maragkakis M., Alexiou P., Grosse I., Hatzigeorgiou A.G. (2012). Functional microRNA targets in protein coding sequences. Bioinformatics.

[bib42] Rehmsmeier M., Steffen P., Hochsmann M., Giegerich R. (2004). Fast and effective prediction of microRNA/target duplexes. RNA.

[bib43] Salmena L., Poliseno L., Tay Y., Kats L., Pandolfi P.P. (2011). A ceRNA hypothesis: the Rosetta Stone of a hidden RNA language?. Cell.

[bib44] Shin C., Nam J.W., Farh K.K., Chiang H.R., Shkumatava A., Bartel D.P. (2010). Expanding the microRNA targeting code: functional sites with centered pairing. Mol. Cell.

[bib45] Su H., Trombly M.I., Chen J., Wang X. (2009). Essential and overlapping functions for mammalian Argonautes in microRNA silencing. Genes Dev..

[bib46] Tang R., Li L., Zhu D., Hou D., Cao T., Gu H., Zhang J., Chen J., Zhang C.Y., Zen K. (2012). Mouse miRNA-709 directly regulates miRNA-15a/16-1 biogenesis at the posttranscriptional level in the nucleus: evidence for a microRNA hierarchy system. Cell Res..

[bib47] Vella M.C., Reinert K., Slack F.J. (2004). Architecture of a validated microRNA:target interaction. Chem. Biol..

[bib48] Wang W.X., Wilfred B.R., Xie K., Jennings M.H., Hu Y.H., Stromberg A.J., Nelson P.T. (2010). Individual microRNAs (miRNAs) display distinct mRNA targeting “rules”. RNA Biol..

[bib49] Yekta S., Shih I.H., Bartel D.P. (2004). MicroRNA-directed cleavage of HOXB8 mRNA. Science.

[bib50] Zhang C., Darnell R.B. (2011). Mapping in vivo protein-RNA interactions at single-nucleotide resolution from HITS-CLIP data. Nat. Biotechnol..

