# Supplementary material for: Mapping the Human miRNA Interactome by CLASH Reveals Frequent Noncanonical Binding
Source: Cell. 2013 Apr 25;153(3):654–65. doi: 10.1016/j.cell.2013.03.043 (PMC3650559; doi:10.1016/j.cell.2013.03.043)
Supplement: Document S1. Tables S1–S6 [file mmc4.pdf]

Table S1. Modifications to the Detailed CLASH Protocol, Related to Figure 1

DATASET		Main modifications to the published detailed protocol
**E1**	A12	• Enzymatic reactions (PNK,TSAP, RNase treatments) performed at 37°C instead of 20°C<br>• 3' linker ligation with T4 ligase 1 instead of T4 ligase 2 truncated K227Q
**E2**	0727	• Inter-molecular ligation performed on IgG-Dynabeads instead of Ni-NTA agarose (hAgo1 not denatured), then hAgo1-RNA complexes eluted with guanidine hydrochloride and loeaded on Ni-NTA agarose<br>• 3' linker ligation with T4 ligase 1 instead of T4 ligase 2 truncated K227Q
**E3**	L1	• 3' linker ligation with T4 ligase 1 instead of T4 ligase 2 truncated K227Q
**E4**	L2	PUBLISHED DETAILED PROTOCOL
**E5**	TA1	• hAgo1 cut out from IgG-Dynabeads with TEV protease instead of guanidine hydrochloride elution<br>• inter-molecular RNA-RNA ligation in solution (hAgo1 not denatured) for 1 hour instead of on Ni-NTA agarose (hAgo1 denatured) for 16 hours, then loading on Ni-NTA agarose
**E6**	TAON	• hAgo1 cut out from IgG-Dynabeads with TEV protease instead of guanidine hydrochloride elution<br>• inter-molecular RNA-RNA ligation in solution for 16 hours (hAgo1 not denatured) instead of on Ni-NTA agarose (hAgo1 denatured) , then loading on Ni-NTA agarose
**E7**		• prepared according to E4 protocol
**E8**		• sample prepared in parallel with E7 on mixed human and yeast lysates according to E4 protocol
**E9**		• prepared according to E6 protocol
**E10**		• sample prepared in parallel with E9 on mixed human and yeast lysates according to E6 protocol

For each of the six CLASH experiments (E1-E6), the table lists the "internal identifier" used in the Supplemental Datasets, and the main differences in experimental protocol as compared to experiment E4. Experiments E7-E10 were used to assess experimental background by addition of yeast lysates to crosslinked HEK cell lysates.

Table S2. CLASH Read Statistics, Related to Figure 1

Table S2A

	ALL DATASETS		DATASET E4	
	Single reads	%	Single reads	%
**microRNA***	21,832,771	20.6	1,722,779	7.6
**mRNA**	20,134,598	19.0	3,767,064	16.5
**tRNA**	40,910,266	38.5	11,596,776	50.9
**rRNA**	13,491,752	12.7	3,709,881	16.3
**snRNA**	1,546,938	1.5	300,695	1.3
**pseudogenes**	957,987	0.9	183,904	0.8
**lincRNA**	519,538	0.5	138,261	0.6
**snoRNA**	489,682	0.5	88,446	0.4
**other**	6,288,134	5.9	1,272,717	5.6
**Total:**	106,171,666		22,780,523	

Table S2B

	All	containing miRNAs	miRNA-mRNA (% of all)
**ALL DATASETS**			
CHIMERIC READS:	1,700,221	563,984	66,304 (3.9%)
collapsed reads	622,721	106,765	28,255 (4.5%)
number of supported interactions	240,135	26,543	18,514 (7.7%)
number of identified miRNAs			399
number of identified mRNAs [ENSG/ENST]			6,959 / 7,390
**PUBLISHED DETAILED PROTOCOL (E4)**			
CHIMERIC READS:	794,729	303,154	29,943 (3.8%)
collapsed reads	290,221	58,337	17,237 (5.9%)
number of supported interactions	110,148	16,386	12,198 (11.1%)
number of identified miRNAs			340
number of identified mRNAs [ENSG/ENST]			5,427 / 5,705

Table S2C

DATASET:	E7	E8	E9	E10
	**HS**	**HS + SC**	**HS**	**HS + SC**
**protocol**	**E4**		**E6**	
number of mapped reads	545,762	680,408	663,349	716,077
**SINGLE READS**				
mapping unambiguously to human transcriptome	98.8%	97.93%	99.03%	99.08%
mapping unambiguously to yeast genome	0.37%	1.45%	0.26%	0.5%
mapping both to human transcriptome and yeast genome	0.83%	0.62%	0.71%	0.41%
**CHIMERIC READS**				
number of miRNA hybrids	1,700	1,581	79	75
number of miRNA hybrids (collapsed reads)	900	938	74	69
number of miRNA-yeast hybrids (collapsed reads)	1	16	0	7
miRNA-yeast hybrids in all miRNA hybrids (collapsed)	0.11%	1.7%	0%	10.1%

(A) Numbers of non-chimeric reads ("single reads") mapping to various RNA species. Sequencing reads were mapped to a custom human transcriptome database using BLAST, as described in detail in Experimental procedures. On average, 70% of all reads could be mapped to this database. *The proportion of sequences mapping to miRNA depends on the size of fragments recovered from the agarose gel in the final stage of cDNA library preparation. In some experiments, including experiment E4, we specifically depleted samples in short cDNAs to increase sequencing depth of longer reads.

(B) Chimeric read statistics. Chimeric reads represent about 2% of all the cDNAs in the CLASH library and about 30% of them correspond to interactions between miRNAs and various RNA species (see also Figure 1D). Identification of chimeric reads and their analysis is described in detail in Experimental Procedures.

(C) Read statistics in the mixed human-yeast lysates experiment. Human and yeast lysates were mixed during CLASH to estimate the background originating from RNA-RNA interactions that can happen after cell lysis. High throughput sequencing reads were mapped to a custom database containing the human transcriptome and yeast genome. HS, experiment performed on human sample; HS + SC, experiment performed on mixed human and yeast sample.

Table S3. Experimentally Validated miRNA Targets Found in CLASH Data Set, Related to Figure 2

miRNA	mRNA	number of chimeric reads supporting interaction	found in experiments:
MIMAT0000062_let-7a	ENSG00000034063_UHRF1	2	E4
	ENSG00000055208_TAB2	16	E3, E4
	ENSG00000077809_GTF2I	1	E5
	ENSG00000100697_DICER1	9	E3, E4
	ENSG00000102531_FNDC3A	1	E3, E4
	ENSG00000105810_CDK6	15	E2
	ENSG00000115760_BIRC6	5	E2, E4
	ENSG00000137309_HMGA1	1	E4
	ENSG00000149948_HMGA2	19	E3, E4
	ENSG00000153048_CARHSP1	2	E4
	ENSG00000163214_DHX57	1	E4
	ENSG00000213516_RBMXL1	1	E4
MIMAT0000063_let-7b	ENSG00000004864_SLC25A13	10	E2
	ENSG00000055208_TAB2	4	E4
	ENSG00000072501_SMC1A	3	E4
	ENSG00000096696_DSP	4	E5, E6
	ENSG00000134824_FADS2	1	E7
	ENSG00000136997_MYC	1	E3
	ENSG00000137309_HMGA1	4	E2
	ENSG00000162063_CCNF	2	E4
	ENSG00000163214_DHX57	2	E3
	ENSG00000164284_GRPEL2	1	E4
	ENSG00000196497_IPO4	2	E4
MIMAT0000064_let-7c	ENSG00000100075_SLC25A1	1	E2
	ENSG00000149948_HMGA2	9	E2, E4
	ENSG00000162063_CCNF	1	E4
MIMAT0000065_let-7d	ENSG00000163214_DHX57	2	E3
MIMAT0000066_let-7e	ENSG00000077809_GTF2I	1	E6
	ENSG00000096696_DSP	2	E4
	ENSG00000129128_SPCS3	2	E4
	ENSG00000134874_DZIP1	3	E4
	ENSG00000142186_SCYL1	1	E4
	ENSG00000145996_CDKAL1	1	E4
	ENSG00000163214_DHX57	2	E3
MIMAT0000067_let-7f	ENSG00000055208_TAB2	4	E4
	ENSG00000149948_HMGA2	2	E4
	ENSG00000159217_IGF2BP1	3	E4
MIMAT0000415_let-7i	ENSG00000164045_CDC25A	1	E6
MIMAT0000068_miR-15a	ENSG00000110092_CCND1	1	E6
	ENSG00000118971_CCND2	17	E2
MIMAT0000069_miR-16	ENSG00000105173_CCNE1	2	E3
	ENSG00000141101_NOB1	1	E6
MIMAT0000070_miR-17	ENSG00000118971_CCND2	1	E3
	ENSG00000124201_ZNFX1	4	E3, E4
	ENSG00000136997_MYC	1	E6
	ENSG00000142192_APP	7	E4
MIMAT0000071_miR-17*	ENSG00000026025_VIM	76	E3, E4, E6
MIMAT0000075_miR-20a	ENSG00000103479_RBL2	1	E6
	ENSG00000140299_BNIP2	3	E4
MIMAT0000082_miR-26a	ENSG00000107864_CPEB3	2	E5, E6
MIMAT0000419_miR-27b	ENSG00000148400_NOTCH1	2	E4
MIMAT0000100_miR-29b	ENSG00000106348_IMPDH1	1	E5
MIMAT0000420_miR-30b	ENSG00000145907_G3BP1	1	E6
MIMAT0000244_miR-30c	ENSG00000090905_TNRC6A	6	E4
	ENSG00000153922_CHD1	1	E4
MIMAT0000692_miR-30e	ENSG00000064042_LIMCH1	1	E4
	ENSG00000114353_GNAI2	1	E5
MIMAT0000103_miR-106a	ENSG00000166483_WEE1	3	E4
MIMAT0000423_miR-125b	ENSG00000108349_CASC3	4	E4
MIMAT0000424_miR-128	ENSG00000106799_TGFBR1	1	E6
MIMAT0000256_miR-181a	ENSG00000111276_CDKN1B	1	E6
MIMAT0000455_miR-185	ENSG00000105810_CDK6	1	E4
MIMAT0000456_miR-186	ENSG00000131016_AKAP12	2	E4
MIMAT0002819_miR-193b	ENSG00000110092_CCND1	6	E4
MIMAT0000226_miR-196a	ENSG00000037965_HOXC8	189	E1, E3, E4, E6
	ENSG00000120068_HOXB8	62	E2, E3, E4
	ENSG00000122592_HOXA7	1	E4
MIMAT0001080_miR-196b	ENSG00000037965_HOXC8	5	E4
	ENSG00000120068_HOXB8	19	E2, E3, E4
MIMAT0000227_miR-197	ENSG00000134569_LRP4	2	E4
	ENSG00000140391_TSPAN3	1	E4
MIMAT0000617_miR-200c	ENSG00000148516_ZEB1	2	E4

MIMAT0000267_miR-210	ENSG00000136003_ISCU	2	E3
MIMAT0000279_miR-222	ENSG00000141510_TP53<br>ENSG00000221914_PPP2R2A	1<br>2	E4<br>E6
MIMAT0000756_miR-326	ENSG00000067225_PKM2	1	E4
MIMAT0005797_miR-1301	ENSG00000026025_VIM	1	E4

A list of experimentally validated targets was compiled from TarBase (Vergoulis et al., 2012) and miRTarBase (Hsu et al., 2011). The overlap between those databases and the CLASH dataset was calculated as described in Experimental Procedures. About 40% of validated interactions are supported by a single chimeric read.

Table S4. Overlap between Targets Identified in CLASH, CLASH Single Read Clusters, PAR-CLIP Clusters, and miRNA Targets Predicted by Common Algorithms, Related to Figure 2

Table S4A

		PAR-CLIP clusters	CLASH single read clusters
	Number of interactions	*15,823*	*28,515*
**CLASH chimeras**	*18,514*	1,596 (9%)	3,066 (17%)
random localization within the same gene	*18,514*	1,125	2,036
random localization within a random gene with similar expression level	*18,514*	540	968
random localization within a random gene	*18,514*	268	439
**CLASH single read clusters**	*28,515*	2,528 (9%)	
random localization within the same gene	*28,515*	1,417	
random localization within a random gene with similar expression level	*28,515*	702	
random localization within a random gene	*28,515*	343	

Table S4B

		matches with CLASH	matches with control	enrichment
	Number of interactions	*6,248*	*6,248*	
**miRanda**	*687,208*	411	29	14.2 ×
**PicTar**	*205,263*	224	9	24.9 ×
**PITA**	*192,255*	195	2	97.5 ×
**RNAhybrid**	*992,584*	310	25	12.4 ×
**TargetScan**	*54,199*	170	5	34.0 ×
**all predictions**	*2,131,509*	802	59	13.6 ×

(A) The overlap between targets identified in CLASH, CLASH single read clusters, PAR-CLIP clusters.

(B) The overlap between CLASH targets, randomized dataset and predictions was calculated using BEDTools (Quinlan and Hall, 2010) as described in Supplemental Experimental Procedures. Enrichment was calculated as fold difference of observed overlap over the overlap expected by chance. For the five prediction methods, CLASH target enrichment ranged from 12- to 97-fold, and the enrichment with all predictions combined was 13.6-fold.

Table S5. Motifs Enriched in CLASH Targets of Individual miRNAs, Related to Figure 4

miRNA	motif start position	motif end position	motif sequence
MIMAT0000089_miR-31	1	7	CTTGCCT
MIMAT0000275_miR-218	1	7	AGCACAA
MIMAT0000692_miR-30e	1	7	GTTTACA
MIMAT0000732_miR-378	1	7	GTCCAGT
MIMAT0004550_miR-30c-2*	1	7	CTCCCAG
MIMAT0000062_let-7a	2	8	CTACCTC
MIMAT0000063_let-7b	2	8	CTACCTC
MIMAT0000064_let-7c	2	8	CTACCTC
MIMAT0000065_let-7d	2	8	CTACCTC
MIMAT0000066_let-7e	2	8	CTACCTC
MIMAT0000067_let-7f	2	8	CTACCTC
MIMAT0000068_miR-15a	2	8	TGCTGCT
MIMAT0000071_miR-17*	2	8	ACTGCAG
MIMAT0000074_miR-19b	2	8	TTTGCAC
MIMAT0000086_miR-29a	2	8	TGGTGCT
MIMAT0000226_miR-196a	2	8	ACTACCT
MIMAT0000255_miR-34a	2	8	CACTGCC
MIMAT0000259_miR-182	2	8	TTGCCAA
MIMAT0000423_miR-125b	2	8	CTCAGGG
MIMAT0000443_miR-125a-5p	2	8	CTCAGGG
MIMAT0000455_miR-185	2	8	TCTCTCC
MIMAT0000681_miR-29c	2	8	TGGTGCT
MIMAT0000759_miR-148b	2	8	TGCACTG
MIMAT0001341_miR-424	2	8	TGCTGCT
MIMAT0002174_miR-484	2	8	GAGCCTG
MIMAT0003880_miR-671-5p	2	8	GGCTTCC
MIMAT0003885_miR-454	2	8	TTGCACT
MIMAT0004672_miR-106b*	2	8	CAGTGCG
MIMAT0004983_miR-940	2	8	CCTGCCT
MIMAT0005577_miR-1226	2	8	GCTGGTG
MIMAT0005797_miR-1301	2	8	AGCTGCA
MIMAT0005825_miR-1180	2	8	GCCGGAA
MIMAT0000069_miR-16	3	9	GTGCTGC
MIMAT0000072_miR-18a	3	9	TGCACCT
MIMAT0000080_miR-24	3	9	ACTGAGC
MIMAT0000084_miR-27a	3	9	CACTGTG
MIMAT0000085_miR-28-5p	3	9	GAGCTCC
MIMAT0000253_miR-10a	3	9	TACAGGG
MIMAT0000254_miR-10b	3	9	TACAGGG
MIMAT0000417_miR-15b	3	9	GTGCTGC
MIMAT0000419_miR-27b	3	9	CACTGTG
MIMAT0000760_miR-331-3p	3	9	CCCAGGG
MIMAT0000761_miR-324-5p	3	9	GGGGATG
MIMAT0002874_miR-503	3	9	CCGCTGC
MIMAT0003887_miR-769-3p	3	9	AGATCCC
MIMAT0004679_miR-296-3p	3	9	CCAACCC
MIMAT0004682_miR-361-3p	3	9	CCTGGGG
MIMAT0004748_miR-423-5p	3	9	TGCCCCT
MIMAT0004945_miR-744	3	9	AGCCCCG
MIMAT0005794_miR-1296	3	9	GGGCCCT
MIMAT0000077_miR-22	4	10	CTGGCAG
MIMAT0000095_miR-96	4	10	TAGTGCC
MIMAT0000261_miR-183	4	10	CAGTGCC
MIMAT0000450_miR-149	4	10	CGGAGCC
MIMAT0000510_miR-320a	4	10	CCCAGCT
MIMAT0000710_miR-365	4	10	AGGGGCA
MIMAT0000750_miR-340*	4	10	CTGAGAC
MIMAT0000753_miR-342-3p	4	10	CTGTGTG
MIMAT0000756_miR-326	4	10	GGCCCAG
MIMAT0002819_miR-193b	4	10	AGGGCCA
MIMAT0004509_miR-93*	4	10	GCTCAGC
MIMAT0004693_miR-330-5p	4	10	GGCCCAG

MIMAT0005584_miR-1229	4	10	GTGGTGA
MIMAT0005792_miR-320b	4	10	CCCAGCT
MIMAT0000762_miR-324-3p	5	11	CCTGGGG
MIMAT0003339_miR-421	5	11	TGTCTGT
MIMAT0004776_miR-505*	5	11	TCCTGGC
MIMAT0004784_miR-455-3p	5	11	CCATGGA
MIMAT0000680_miR-106b	6	12	GTCAGCA
MIMAT0003283_miR-615-3p	6	12	CCCAGGC
MIMAT0004504_miR-31*	6	12	GTTGGCA
MIMAT0004680_miR-130b*	6	12	CAGGGAA
MIMAT0004697_miR-151-5p	6	12	TGAGCTC
MIMAT0000087_miR-30a	7	13	GAGGATG
MIMAT0000245_miR-30d	7	13	GGGGATG
MIMAT0003886_miR-769-5p	7	13	CCCAGAG
MIMAT0000731_miR-378*	8	14	CCTGGAG
MIMAT0004978_miR-935	8	14	GAAGCGG
MIMAT0000078_miR-23a	9	15	CCCTGGC
MIMAT0000279_miR-222	9	15	TAGCCAG
MIMAT0000418_miR-23b	9	15	CCCTGGC
MIMAT0005891_miR-1303	9	15	GACCCCG
MIMAT0000082_miR-26a	10	16	TCCTGGA
MIMAT0000757_miR-151-3p	10	16	AGGAGCT
MIMAT0000764_miR-339-5p	10	16	TCCTGGA
MIMAT0002876_miR-505	10	16	CCAGCAA
MIMAT0003888_miR-766	10	16	GCTGTGG
MIMAT0000104_miR-107	11	17	CCCTGTA
MIMAT0000256_miR-181a	11	17	CGACAGC
MIMAT0001340_miR-423-3p	11	17	GGGCCTC
MIMAT0002891_miR-18a*	11	17	GGAGCAC
MIMAT0003218_miR-92b	11	17	CCGGGAC
MIMAT0000103_miR-106a	12	18	TGCACTG
MIMAT0000227_miR-197	12	18	GGTGGAG
MIMAT0000278_miR-221	12	18	CCAGCAG
MIMAT0000703_miR-361-5p	12	18	CCCTGGA
MIMAT0004950_miR-877*	12	18	GGAGGAG
MIMAT0015041_miR-1260b	12	18	TGGTGGC
MIMAT0000081_miR-25	13	19	GACCGAG
MIMAT0000092_miR-92a	13	19	GGCCGGG
MIMAT0000244_miR-30c	13	19	AGAGTGT
MIMAT0000093_miR-93	14	20	CCTGCAC
MIMAT0003266_miR-598	14	20	ACGATGA
MIMAT0000070_miR-17	15	21	ACCTGCA
MIMAT0000088_miR-30a*	15	21	CTGCAAA
MIMAT0000075_miR-20a	16	22	TACCTGC
MIMAT0000098_miR-100	16	22	CACAAGT
MIMAT0000440_miR-191	16	22	AGCTGCT

For the discovery pipeline, see Figure 4 and Supplemental Experimental Procedures.

Table S6. List of Oligonucleotides, Related to Experimental Procedures

PRIMER:	SEQUENCE:	NOTES:
**Primers for Ago1 cloning**		
Ago1_F	TACAAAGCTTGGAGGTATGGAAGCGGGACCCTCGG	
Ago1_R	GCTGAATTCGCCTTCAAGCGAAGTACATGG	
**CLASH linkers and primers**		
3' linker		
miRCat-33 linker (IDT)	AppTGGAATTCTCGGGTGCCAAG/ddC/	
5' linkers (barcode marked red)		
E1: L5_Solexa	invddT-GTTCArGrArGrUrUrCrUrArCrArGrUrCrCrGrArCrGrArUrC-OH	used in experiment E1
E2: L5a	invddT-ACACrGrArCrGrCrUrCrUrUrCrCrGrArUrCrUrArC-OH	used in experiment E2
E3: L5c	invddT-ACACrGrArCrGrCrUrCrUrUrCrCrGrArUrCrUrGrA-OH	used in experiment E3
E4: L5e	invddT-ACACrGrArCrGrCrUrCrUrUrCrCrGrArUrCrUrCrArCrArGrC-OH	used in experiment E4
E5: L5Aa	invddT-ACACrGrArCrGrCrUrCrUrUrCrCrGrArUrCrUrNrNrNrUrArArGrC-OH	used in experiment E5
E6, E10: L5Ab	invddT-ACACrGrArCrGrCrUrCrUrUrCrCrGrArUrCrUrNrNrNrArUrUrArGrC-OH	used in experiments E6 and E10
E7: L5Cc	invddT-ACACrGrArCrGrCrUrCrUrUrCrCrGrArUrCrUrNrNrNrArCrTrCrArGrC-OH	used in experiment E7
E8: L5Cd	invddT-ACACrGrArCrGrCrUrCrUrUrCrCrGrArUrCrUrNrNrNrGrArCrTrTrArGrC-OH	used in experiment E8
E9: L5Ac	invddT-ACACrGrArCrGrCrUrCrUrUrCrCrGrArUrCrUrNrNrNrGrCrGrCrArGrC-OH	used in experiment E9
PCR primers		
miRCat-33 primer (IDT)	CCTTGGCACCCGAGAATT	primer for RT
PE_miRCat_PCR	CAAGCAGAAGACGGCATACGAGATCGGTCTCGGCATTCCTGGCCTTGGCACCCGA GAATTCC	library amplification
P5	AATGATACGGCGACCACCGAGATCTACACTCTTTCCCTACACGACGCTCTTCCGAT CT	library amplification
**Oligonucleotides for creating reporter vectors**		
miR-92a-1xS+M_F	TACTCTCGAGACAGGCCGGGTTCTGTGCAATATATGCGGCCGCATAT	oligo with miR-92a full binding site
miR-92a-1xS+M_R	ATATGCGGCCGCATATATTGCACAGAACCCGGCCTGTCTCGAGAGTA	oligo with miR-92a full binding site
miR-92a-1xseed_F	ATATCTCGAGTAAACTAATTGTGTGCAATATATGCGGCCGCATTA	oligo with miR-92a seed
miR-92a-1xseed_R	TAATGCGGCCGCATATATTGCACACAATTAGTTTACTCGAGATAT	oligo with miR-92a seed
miR-92a-1xmotif_F	GCAACTCGAGGGGCCGGGTTCATACGTTCTAGCGGCCGCCCT	oligo with miR-92a motif
miR-92a-1xmotif_R	AGGGCGGCCGCTAGAACGTATGAACCCGGCCCCTCGAGTTGC	oligo with miR-92a motif
PCGF5_XhoI_For	GCTACTCGAGCAACAAATAGCCTTCCTCTG	3'UTR PCR; ENST00000336126
PCGF5_NotI_Rev	ATTAGCGGCCGCTTGTCAAAGTTGGCTGTATG	3'UTR PCR; ENST00000336126
OSBPL8_XhoI_For	ACGACTCGAGCTACCATTGAATCAGTGAACTAG	3'UTR PCR; ENST00000261183
OSBPL8_NotI_Rev	ATTAGCGGCCGCTTGGCCTATGTTATGAACC	3'UTR PCR; ENST00000261183
RFFL_XhoI_For	CTTCCTCGAGCTTTGAATCCTCCCTTCTG	3'UTR PCR; ENST00000315249
RFFL_NotI_Rev	ATTAGCGGCCGCGGGTTCACACCATTCTCC	3'UTR PCR; ENST00000315249
MAPRE1_XhoI_For	CTTACTCGAGGCCCTGAGATGTTACAGTTG	3'UTR PCR; ENST00000375571
MAPRE1_NotI_Rev	ATTAGCGGCCGCCACAGAGCATGCACTACAC	3'UTR PCR; ENST00000375571
OSBPL2_XhoI_For	CGTACTCGAGCTGAGTTCGCGGAGATAG	3'UTR PCR; ENST00000358053
OSBPL2_NotI_Rev	ATTAGCGGCCGCACACCACTGTAAGTAGATTC	3'UTR PCR; ENST00000358053
OSBPL8_PCR_mut_F	GCGCACTAGTATCCAGTGTTCAAGTTGGTTTGG	Primer for mutagenesis
OSBPL8_PCR_mut_R	CGCGACTAGTTAAATCAGATCTTTCTAGTTCACTG	Primer for mutagenesis
**Primers for qRT-PCR**		
miR-92a CLASH targets – motif		
SCLY_FW	GCTGCGGGAAACACAACT	target: ENST00000254663, probe: 55
SCLY_REV	GGGGAGTCGTTGCATTATAGTC	target: ENST00000254663, probe: 55
RPL3_6_Fw	CAAGTCATCCGTGTCATTGC	target: ENST00000216146, probe: 6
RPL3_6_REV	ACAGTGCCTCCGTTCACCT	target: ENST00000216146, probe: 6
RPS5_53_FW	CTCAGGCTGTGTTCTCAGGAT	target: ENST00000196551, probe: 53
RPS5_53_REV	TCCACTTCCCAAAGAGCTTG	target: ENST00000196551, probe: 53
KAT2A_25_FW	TCCTCACTCACTTCCCCAAA	target: ENST00000225916, probe: 25
KAT2A_ 25_REV	TTGGAGAGTTTGCCCCATAG	target: ENST00000225916, probe: 25
MAPRE1_ 62_FW	GACGAGGCAGCTGAGTTGA	target: ENST00000375571, probe: 62
MAPRE1_ 62_REV	TTCCGAAGTAGAAATCCCTCTCT	target: ENST00000375571, probe: 62
C9orf7_FW	GGGTGCTGTACGGACTCTCT	target: ENST00000316948, probe: 34
C9orf7_REV	CGCGAGCTTCTCCTCATC	target: ENST00000316948, probe: 34
C1orf114_FW	AACAGAAGGCCGGGAAAG	target: ENST00000367805, probe: 53
C1orf114_REV	TGTCTAGTTCTCTCTCTGACAGCTTG	target: ENST00000367805, probe: 53
MSL3_79 _FW	GTGCCACAAGCACTAACAGG	target: ENST00000337339, probe: 79
MSL3_79 _REV	GATTCAACAAAGGCGGACTG	target: ENST00000337339, probe: 79
CCT7_83_FW	CAGGAGGGCCATCAAGAAT	target: ENST00000258091, probe: 83
CCT7_83_REV	GCAGGTACTTGGAGAGTTCCAT	target: ENST00000258091, probe: 83
CAPRIN1_68_FW	GGCAGAAACACAGTTCACCA	target: ENST00000341394, probe: 68
CAPRIN1_68_REV	AGGTTGCTGCTGGAGTGAAT	target: ENST00000341394, probe: 68

TRAP1_9 _FW	AGACCAATGCCGAGAAAGG	target: ENST00000246957, probe: 9
TRAP1_9 _REV	TCCTGTGTCATCCCGATACC	target: ENST00000246957, probe: 9
miR-92a CLASH targets – seed		
CUX1_36_FW	GACTCTGCCAGGTGGATGTT	target: ENST00000292535, probe: 36
CUX1_36_REV	GTTCGCCAATACCGTTGC	target: ENST00000292535, probe: 36
TANC2_84_FW	CGAGAGCAAGGGCAAAAC	target: ENST00000424789, probe: 84
TANC2_84_REV	GCCTCGTTCAGGTCCTCTAA	target: ENST00000424789, probe: 84
FNIP1_89_FW	CGTCAGTGTCTGATGTCAGATTT	target: ENST00000307968, probe: 89
FNIP1_89_REV	AGCTTCTGCTATTGGTTCATCC	target: ENST00000307968, probe: 89
EHBP1_FW	CGACAGCGGGATTAGAGC	target: ENST00000354487, probe: 9
EHBP1_REV	GATGAGTGGGAAGGGGTTC	target: ENST00000354487, probe: 9
RASAL2_21_FW	TGCCTTAGTGCACATTCTTCA	target: ENST00000462775, probe: 21
RASAL2_21_REV	CCACCTCAGACATCACCAAG	target: ENST00000462775, probe: 21
RBM10_68_FW	GGCAAGCATGACTATGACGA	target: ENST00000377604, probe: 68
RBM10_68_REV	GGGAGGCCTCGTAGGAAT	target: ENST00000377604, probe: 68
PCMTD1_FW	AAGTACAATGGTGGGCTTAATTTT	target: ENST00000360540, probe: 31
PCMTD1_REV	AGCTCTCCAGTTTTTCCTTGG	target: ENST00000360540, probe: 31
RORA_2_FW	TACATGTGAAGGCTGCAAGG	target: ENST00000335670, probe: 2
RORA_2_REV	GTTCTTCTGACGAGGACAGGA	target: ENST00000335670, probe: 2
SRPR_5_FW	GATGGGGACGTTCAGCAC	target: ENST00000332118, probe: 5
SRPR_5_REV	CCGGAGCATGTCTACACGA	target: ENST00000332118, probe: 5
miR-92a negative controls		
UBXN8_FW	GGTTGTTGGCATTTTCTTCC	target: ENST00000265616, probe: 56
UBXN8_REV	GCAAAAGAAAATCCTTGATTCC	target: ENST00000265616, probe: 56
TPRKB_FW	GCAAACAAAGCAGTTCACCTC	target: ENST00000272424, probe: 48
TPRKB_REV	AAGTGTCATTTGCTGAGATACCAA	target: ENST00000272424, probe: 48
SIP1_FW	GGTGAAAAGTTATGTGCTGACG	target: ENST00000250379, probe: 5
SIP1_REV	CCAATTACTCAGATATTCCAAGACAC	target: ENST00000250379, probe: 5
ADK_FW	TTCAATTAAAGTGGCTCAGTGG	target: ENST00000286621, probe: 5
ADK_REV	TCTATCCCAATGCATCCAAAA	target: ENST00000286621, probe: 5
ADAL_FW	TGAAGTTAGCATTGCATCTTTCAG	target: ENST00000305605, probe: 38
ADAL_REV	ATGTTCCATGCCCGATTCT	target: ENST00000305605, probe: 38
Other miR-92a target		
12652-2_lincRNA_F	GCAAAAATCCCTCATGCAGT	target: ENST00000500949, probe: 4
12652-2_lincRNA_R	TCAAACATCCAAGTATCCACCTT	target: ENST00000500949, probe: 4
**miRNA inhibitors synthesized by IDT**		
NC5 AMO	mC/ZEN/mGmCmGmAmCmUmAmUmAmCmGmCmGmCmAmAmUmAm UmGmG/ZEN/mU	Negative control
Hsa-miR-92a-3p ZEN AMO	mA/ZEN/mCmAmGmGmCmCmGmGmGmAmCmAmAmGmUmGmCmA mAmU/ZEN/mA	miR-92a inhibitor
